# Dynein‐Powered Cell Locomotion Guides Metastasis of Breast Cancer

**DOI:** 10.1002/advs.202302229

**Published:** 2023-09-19

**Authors:** Yerbol Tagay, Sina Kheirabadi, Zaman Ataie, Rakesh K. Singh, Olivia Prince, Ashley Nguyen, Alexander S. Zhovmer, Xuefei Ma, Amir Sheikhi, Denis Tsygankov, Erdem D. Tabdanov

**Affiliations:** ^1^ Department of Pharmacology Penn State College of Medicine The Pennsylvania State University Hershey PA 17033 USA; ^2^ Department of Chemical Engineering The Pennsylvania State University University Park PA 16802 USA; ^3^ Department of Obstetrics & Gynecology Gynecology Oncology University of Rochester Medical Center Rochester NY 14642 USA; ^4^ Center for Biologics Evaluation and Research U.S. Food and Drug Administration Silver Spring MD 20903 USA; ^5^ Department of Biomedical Engineering The Pennsylvania State University University Park PA 16802 USA; ^6^ Wallace H. Coulter Department of Biomedical Engineering Georgia Institute of Technology and Emory University Atlanta GA 30332 USA; ^7^ Penn State Cancer Institute Penn State College of Medicine The Pennsylvania State University Hershey PA 17033 USA

**Keywords:** contact guidance, dynein, granular hydrogel, mechanobiology, microtubules, motility

## Abstract

The principal cause of death in cancer patients is metastasis, which remains an unresolved problem. Conventionally, metastatic dissemination is linked to actomyosin‐driven cell locomotion. However, the locomotion of cancer cells often does not strictly line up with the measured actomyosin forces. Here, a complementary mechanism of metastatic locomotion powered by dynein‐generated forces is identified. These forces arise within a non‐stretchable microtubule network and drive persistent contact guidance of migrating cancer cells along the biomimetic collagen fibers. It is also shown that the dynein‐powered locomotion becomes indispensable during invasive 3D migration within a tissue‐like luminal network formed by spatially confining granular hydrogel scaffolds (GHS) made up of microscale hydrogel particles (microgels). These results indicate that the complementary motricity mediated by dynein is always necessary and, in certain instances, sufficient for disseminating metastatic breast cancer cells. These findings advance the fundamental understanding of cell locomotion mechanisms and expand the spectrum of clinical targets against metastasis.

## Graphical abstract

Breast cancer cells utilize a complementary metastatic locomotion mechanism in the absence of actomyosin contractility via dynein‐driven sliding of the microtubules in respect to the F‐actin cytoskeleton and cell‐collagen adhesions in the adhesion‐guided ‘dendritic’‐like protrusions. Either actomyosin‐ or dynein‐driven cell motility is sufficient for carcinoma cell migration along the artificial anisotropic collagen ‘fibers’, indicating mutually compensating, dynamically balanced metastasis mechanism.

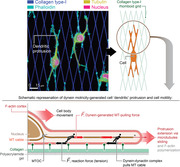

## 1 Introduction

Metastasis is a process of migratory exit of cancer cells from the primary tumor, followed by the invasion of cancer cells into the healthy tissues and formation of secondary tumors. Metastasis is the leading cause of mortality among cancer patients. For metastatic cells, migration in solid tissues represents a confined locomotion within and along the available intercellular luminal or interstitial spaces.^[^ ^1^ ^]^ Similarly, the confined migration of cancer cells in three‐dimensional (3D) collagen‐rich matrices at the tumor‐stroma interfaces occurs via paths of least resistance and along the anisotropically aligned collagen fibers,^[^ ^2^ ^]^ which is known as “contact guidance”.

Most metastatic cells display a reduced actomyosin contractility in confinement, followed by cell “fluidization”.^[^ ^3^, ^4^, ^5^, ^6^, ^7^, ^8^ ^]^ Consequently, the metastatic dissemination of cancer cells poses a question on the sources of alternative mechanical forces that could facilitate the myosin‐independent locomotion of cancer cells during their breakout from the primary tumor^[^ ^9^, ^10^, ^11^ ^]^ and infiltration through the blood vessel walls into the healthy tissues.^[^ ^12^ ^]^ Finding the source of myosin‐independent cell motricity requires a more detailed mechanobiological model of cancer cell migration that could explain the motility of cancer cells in various conditions, including migration of cells with reduced actomyosin contractility.

The growing body of evidence suggests that microtubules and microtubule‐associated motors^[^ ^13^, ^14^, ^15^ ^]^ are directly involved in the locomotion of cancer cells. For example, migration of mesenchymal‐like cancer cells on the collagen‐coated surfaces may depend on the activity of cytoplasmic dynein motors upon reduction of the actomyosin contractility,^[^ ^16^ ^]^ with the latter displaying the instances of interference with the dynein‐dependent cell motility. Similar observations are reported for cancer cells within the fibrous 3D collagen matrices, where mesenchymal‐like cancer cells develop “dendritic”‐like cell protrusions along the collagen fibers in a dynein‐dependent manner.^[^ ^17^ ^]^ Moreover, the pharmacological targeting of mechanically antagonistic dynein and kinesin‐1 motors in cancer cells interferes with the organization of microtubules, cell shape, and contact guidance, as demonstrated by spatial alignment of cancer cells on either topographic or flat anisotropic adhesion cues.^[^ ^14^ ^]^ These findings highlight the potential importance of the microtubules and microtubule‐associated motors for the cell's ability to sense and interact with its environments.

Mechanistically, microtubules may serve as an important cell‐scaffolding structure that enables cell alignment to the nano‐textures via direct mechanical and steric interactions of microtubules with the 3D features of the microenvironment.^[^ ^13^ ^]^ Moreover, the mechanical role of microtubules was also demonstrated for stable detyrosinated microtubules that have a strut‐like mechanical function in the cyclically contracting myofibrils within cardiac myocytes.^[^ ^18^ ^]^ These observations suggest that the role of microtubules and microtubule‐associated motors may span beyond intracellular trafficking, signaling, and cell division.

Here, we report that dynein motricity is necessary to support the migration of cancer cells upon inhibition of actomyosin contractility. Specifically, we show that while non‐muscle myosin II (NMII) contractility and dynein motricity coexist, each group of motors is able to maintain migration of cancer cells along the 2D biomimetic collagen “fibers” network. We also show that cancer cells require the simultaneous activity of dynein and myosin motors during the confined 3D migration in gelatin‐based granular hydrogels, suggesting, for the first time, a fundamental mechanobiological difference between confined and unconfined modes of metastasis.

## 2 Results

### 2.1 Upregulated Expression of Dynein and Dynactin is Linked to Breast Cancer Aggressiveness

**Figure 1:**
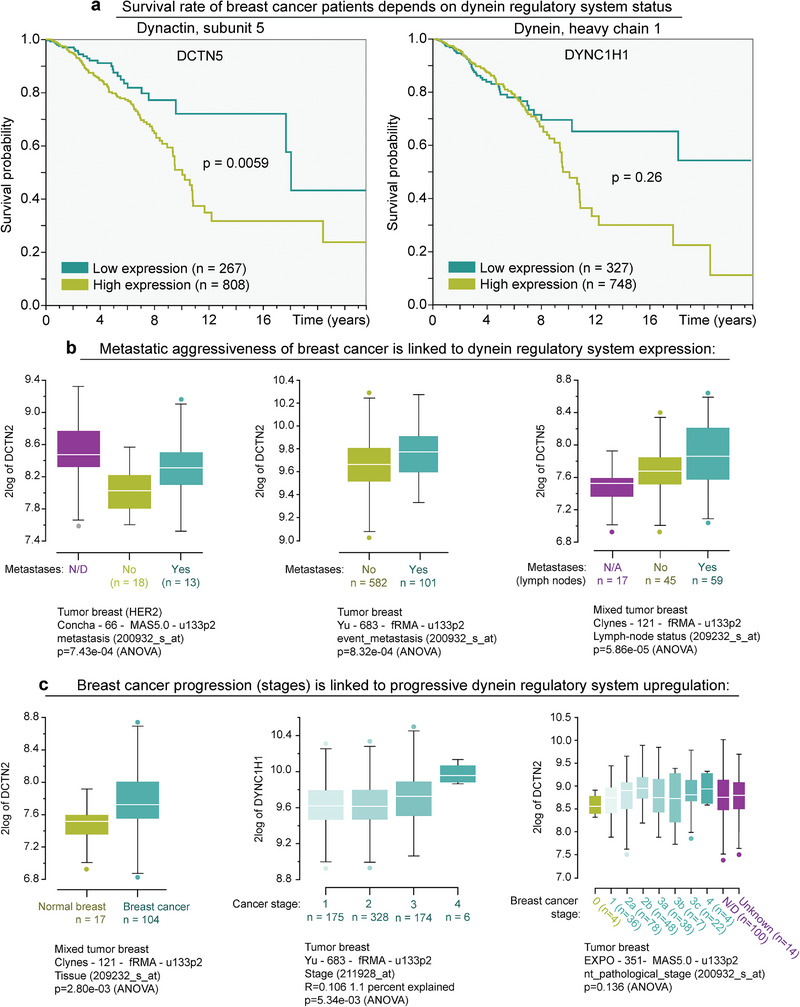
Upregulation of dynein and its regulatory cofactor dynactin's subunits are linked to the higher mortality rate and metastatic aggressiveness in breast cancer patients. a) Dynactin's subunit 5 (DCTN5, left) and cytoplasmic dynein's heavy chain 1 (DYNC1H1, right) upregulation indicates a lower survival rate of breast cancer patients. b) Metastatic aggressiveness of breast cancer is linked to overexpression of dynactin subunits 2 (DCTN2, left and center) and 5 (DCTN5, right). c) Breast cancer stage progression is linked to the progressive upregulation of the dynein regulatory system. The binary difference in DCTN2 expression in normal breast versus breast cancer tissues (left); progressive increase of the dynein heavy chain 1 overexpression across first (non‐metastatic) through fourth (metastatic) breast cancer stages (DYNC1H1, center); and progressive upregulation of the dynactin subunit 2 across various stages of breast cancer stages (DCTN2, right). Note that breast cancer TCGA data available at “The Human Protein Atlas” was analyzed using the built‐in tools to obtain survival curves shown in Figure 1a (left, right). Figures 1b,c were output by the MegaSampler tool of R2‐Genomics Analysis and Visualization Platform that viewed genes in more than one breast cancer microarray databases. The names of the databases analyzed by the MegaSampler tool are provided in the respective figures.

Several recent studies indicate that dysregulation of microtubule motors, such as dynein and its dynactin cofactors, is linked to cancer progression.^[^ ^19^, ^20^, ^21^, ^22^, ^23^, ^24^ ^]^ The bioinformatic analysis, using the open access “The Human Protein Atlas” database,^[^ ^25^ ^]^ and R2‐Genomics and Visualization Platform^[^ ^26^ ^]^ shows that elevated expression of dynactin's subunit 5 (**Figure** **1**a, left) and dynein's heavy chain 1 (Figure 1a, right) could be used as a predictive marker of a substantially lower survival rate for breast cancer patients.^[^ ^26^ ^]^

The upregulated expression of dynactin's subunit 2 (DCTN2) and subunit 5 (DCTN5) predicts the metastatic aggressiveness of breast cancer in patients (Figure 1b).^[^ ^27^ ^]^ Analysis of the progression of breast cancer metastatic aggressiveness also conveys an incremental correlation between cancer stage progression and the overexpression of dynactin's subunit 2 (DCTN2) and dynein heavy chain 1 (DYNC1H1) (Figure 1c).^[^ ^27^ ^]^ Thus, the analyzed data motivates additional experiments for unraveling the potential role of microtubule motors in breast cancer metastasis.

### 2.2 Biomimetic Grid Emulates Tumor‐Associated Collagen Signatures for Metastasis Studies

**Figure 2:**
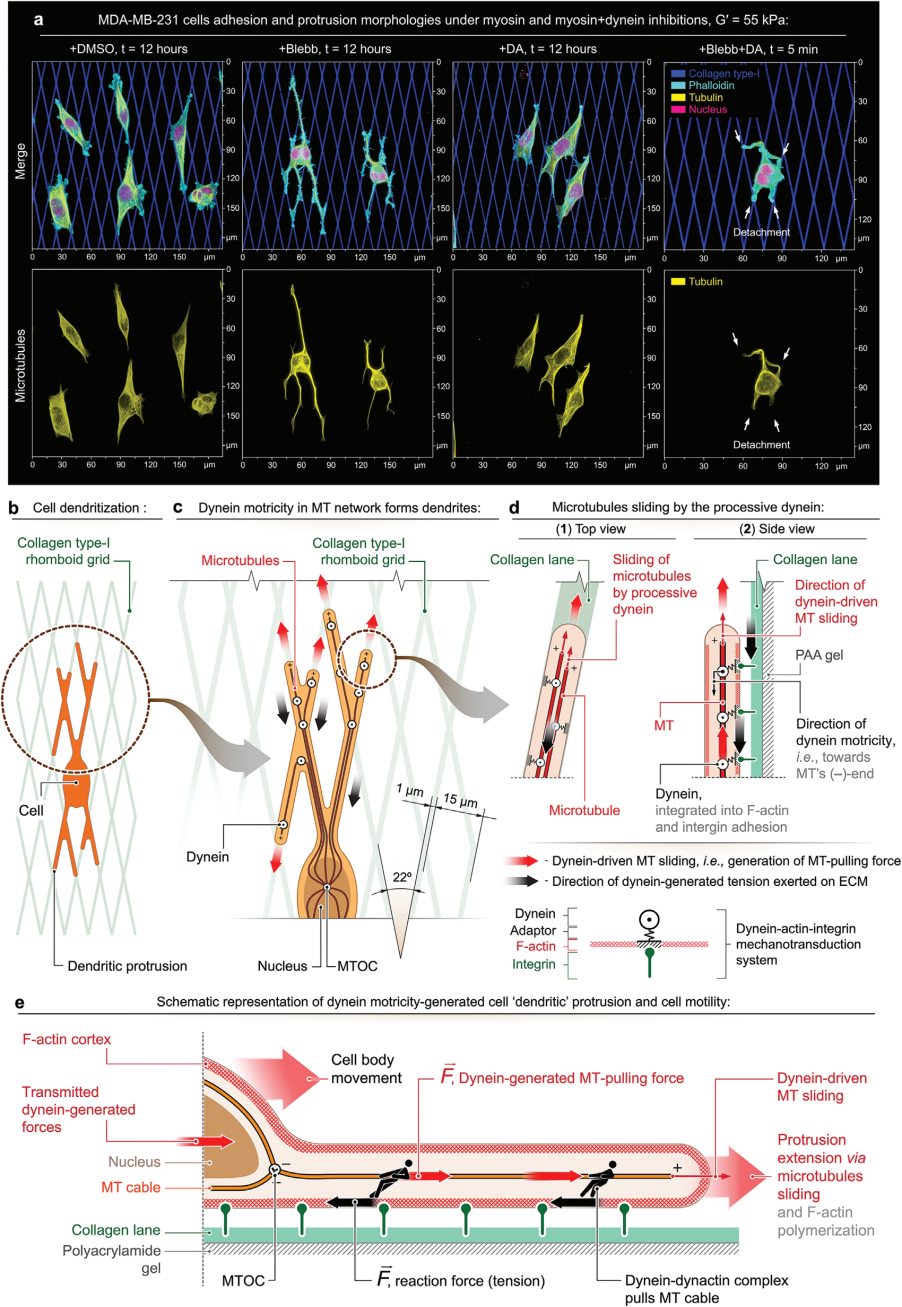
Adhesion and protrusion of metastatic MDA‐MB‐231 cells along the collagen type‐1 patterns depend on actomyosin contractility and complementary microtubule‐dynein tension‐generating system. a) MDA‐MB‐231 morphological reorganization in response to adhesion and protrusion along the anisotropic collagen “fibers” (i.e., rhomboid grid) in the control conditions (+DMSO), during the suppression of actomyosin contractility with blebbistatin (+Blebb), during dynarrestin‐induced dynein‐microtubules dissociation (+DA), and upon dynein‐MT dissociation with dynarrestin during a prolonged suppression of actomyosin contractility with blebbistatin (+Blebb+DA). The shown microscopy images correspond to the rigid (*G*′ = 55 kPa) collagen‐1 grids. Cells protrusion on the soft collagen grids (*G*′ = 8.6 kPa) in the same treatment conditions are shown in Figure S2a, Supporting Information. Note the “dendritic” architecture of cells during Blebbistatin‐induced suppression of actomyosin contractility (+Blebb) and a high level of structural conformity of cell protrusions to the underlying collagen grid. Structural conformity indicates protrusions’ adhesion to the collagen grid throughout their lengths. b) Schematic view of the dendritized MDA‐MB‐231 cell on the collagen rhomboid grid during actomyosin contractility suppression with blebbistatin. c,d) Suggested schematic representation of the collagen‐guided elongation of dendritic‐like protrusions along the rhomboid grid. Note that microtubules structurally scaffold and support the “dendritic”like protrusions. c) Direction of protrusive growth of cell's “dendritic”‐like projections (red arrows) guided along the collagen lanes. Note the structural characteristics of the collagen rhomboid grid: two sets of 1‐µm‐wide 15‐µm‐pitched parallel collagen lanes, crisscrossed into a rhomboid grid at 22° angle. d) “Dendritic”‐like protrusions extend along the collagen “fibers” via the activity of dynein motors that slide the protrusions' structural core (microtubules) towards their plus‐ends. The mechanical coupling between the microtubules to the guiding collagen lanes is enabled via dynein motors embedded into the F‐actin cytoskeleton that, in turn, is coupled to the ECM via the adhesion apparatus (e.g., integrin system). e) Schematic representation of the cell motility, driven by force distribution in cell's “dendritic”‐like protrusions generated via dynein motricity during reduced actomyosin contractility. The balance between the dynein‐generated MT pulling forces and the arising reaction forces that constitute tension are shown as a “tug‐of‐war” driven by the dynein motors.

To study metastasis of breast cancer cells, we initially tested two biomimetic substrates, designed to resemble the fibrous structure of extracellular matrix (ECM) for a directed cell migration. One design is a single set of one‐dimensional (1D) 1 µm‐wide parallel collagen‐1 lanes, spaced by a 15 µm‐wide pitch, while another design represents two such sets of lanes, arrayed into the two‐dimensional (2D) rhomboid grids, crisscrossed at 22° angle (**Figure** **2**a–c). The selected angle of crisscrossed biomimetic collagen “fibers” is within the range reported for the anisotropically oriented yet structurally noisy tumor‐associated collagen signatures (TACS‐3)^[^ ^2^ ^]^ located at metastatically active tumor‐stroma interfaces.^[^ ^2^, ^10^ ^]^

The side‐by‐side comparison of metastatic MDA‐MB‐231 cell migration along the 1D collagen lanes and 2D collagen grids with rigidity shear modulus *G*′ = 8.6 kPa (Figures S1a,b, and Movie S1, Supporting Information) shows that both underlying collagen patterns direct cell migration, enabling contact guidance along the adhesion cues (Figure S1c, Supporting Information). However, MDA‐MB‐231 cells migrating on the 1D collagen lanes demonstrate a heterogeneous, bi‐modal distribution of cell speeds compared to the single mode of migration speeds on the 2D collagen grids (Figure S1d, Supporting Information). Notably, the speed modes for cells migrating along the 1D collagen lanes are both above and below the single speed mode for cells on the 2D rhomboid grids. Additionally, the analysis of the overall cell displacement (mean squared displacement, i.e., MSD) shows a lower motility efficiency in cells on 1D collagen lanes, compared to 2D collagen grids (Figure S1e, Movie S1, Supporting Information), despite a lower directionality ratio of cells on the 2D collagen grids (Figure S1f, Supporting Information).

The heterogeneity of locomotion and lower efficiency of motility of metastatic cells on the 1D collagen lanes is consistent with the previously reported studies, highlighting the formation of 1D cell protrusions and 1D migration as a special case of cell mechanobiology.^[^ ^13^, ^15^, ^28^, ^29^ ^]^ Notably, unlike 2D collagen grids, the 1D collagen lanes naturally induce a highly symmetric spindle‐like cell morphology of MDA‐MB‐231 cells,^[^ ^13^ ^]^ often resulting in the balanced “tug‐of‐war” cell state and, effectively, stalling cell migration (Figure S1b, Supporting Information). While the symmetric distribution of opposing protrusive forces may contribute to the migration stalling of some of the cells on the 1D collagen lanes, the higher frequency of cell‐cell collisions of the other cells migrating in opposing directions along the same lane may also obstruct migration (Movie S1, Supporting Information), as opposed to the cells migrating on the 2D collagen grids that provide cells with a way around each other. Indeed, analysis of the angular distribution of cell 1 min‐long displacements indicates that MDA‐MB‐231 cells migrating along the sparse 1D collagen lanes are more likely to remain on their original lanes. On the other hand, the MDA‐MB‐231 cells migrating along the 2D collagen grids demonstrate substantial directional flexibility by also being able to move transversely to the anisotropy axis of the rhomboid grids and bypass the obstructing cells (Figure S1g, Supporting Information).

Therefore, to study a potential role for microtubule motors in breast cancer metastasis, we choose 2D rhomboid collagen grids over the common design of 1D parallel collagen lanes. Our choice of design is dictated by the necessity to avoid the identified artifacts of the 1D cell migration and by our aim to separate the respective contributions of myosin‐ and dynein‐driven mechanisms of cell migration. Specifically, the seminal mechanobiology studies have established that adhesion and crawling of mesenchymal cells along the 1D adhesion cues, such as bundled, anisotropically aligned collagen fibers or micropatterns of isolated adhesion lanes, often represent a special case of cell migration that generally does not critically require actomyosin contractility.^[^ ^13^, ^28^, ^29^, ^30^ ^]^ Thus, we conclude that 2D crisscrossed collagen rhomboid grids are better suited for deciphering the contributing roles of myosin and dynein motors in the migration of metastatic breast cancer cells.

To test the adhesion, protrusion formation, spreading, and migration of metastatic MDA‐MB‐231 cells, we use soft, *G*′ = 8.6 kPa, (Figure S1a, Supporting Information) and rigid, *G*′ = 55 kPa, (Figure 1a) collagen grids. The soft and rigid collagen grids represent the mechanical range measured in vivo for human breast structures, with shear moduli *G*′ fluctuating from 5 kPa in fatty tissue to 50 kPa in breast parenchyma,^[^ ^31^ ^]^ and for human breast cancer lesions, with moduli fluctuating from 10 kPa through 42 kPa.^[^ ^32^ ^]^

### 2.3 Low Actomyosin Contractility Reveals that Cell Adhesion and Protrusion Depend on Dynein

Using the biomimetic collagen grids, we compare four conditions: control cells treated with DMSO (Figures S2a and 2a, Supporting Information), cells with low actomyosin contractility due to the blebbistatin treatment for 12 h (Figures S2a and Figure 2a, Supporting Information), cells with low dynein activity, i.e., treated with dynarrestin^[^ ^14^, ^33^ ^]^ for 12 h (Figures S2a and Figure 2a, Supporting Information), and cells after subsequent 12‐h‐long blebbistatin treatment, followed by co‐treatment with dynarrestin (Figures S2a and Figure 2a, Supporting Information).

Actomyosin contractility is a principal mechanism of cell migration.^[^ ^34^ ^]^ However, its inhibition by targeting non‐muscle myosin II activity with blebbistatin^[^ ^35^, ^36^ ^]^ does not prevent adhesion or protrusive spreading of metastatic MDA‐MB‐231 cells on the 2D collagen rhomboid grids. Moreover, the compact “polygonal” shape of cells on the collagen grids in control conditions indicates their limited protrusiveness along the collagen grid (Figures S2a, 2a, and Movie S2, Supporting Information) compared to the increased lengths of protrusion along the same pattern for blebbistatin‐treated cells (Figures S2a, 2a, and Movie S3, Supporting Information). The morphological analysis of MDA‐MB‐231 cell widths and lengths in response to all utilized treatments is shown in Figures S2b and S2c, Supporting Information. The analysis shows a substantial increase in the cell lengths and widths in response to blebbistatin treatment, attributed to the extensive formation of “dendritic”‐like cell protrusions along the underlying grids.

We note that pairwise comparison of corresponding cell protrusion dimensions (lengths and widths) between soft and rigid collagen grids for each individual type of treatment displays a marginal, often statistically insignificant difference (Figure S2c, Supporting Information), demonstrating that blebbistatin treatment induces similar effects in cells on soft and rigid grids. These data are consistent with the previous reports showing that mechanobiological and migratory responses of mesenchymal cells to the highly structured, fibril‐like, spatially anisotropic adhesion configurations are principally different from cell responses reported for the continuous, 2D, solid, and uniform adhesive surface routinely used for cell adhesion and migration studies.^[^ ^28^ ^]^ For example, MDA‐MB‐231 and MDA‐MB‐468 cells display a limited, if any, response to the mechanical rigidity of the 1D anisotropic fibril‐like collagen‐1 adhesion signals.^[^ ^13^, ^15^ ^]^

The live observation of cell response to the blebbistatin treatment in MDA‐MB‐231 cells adhered to the collagen rhomboid grids identifies an initial, yet transient, stage of partial cell retraction that lasts for 11 min. The retraction stage is then quickly followed by the sustained protrusive spreading with a formation of “dendritic”‐like cell protrusions and cell crawling along the collagen grids (Movie S4, Figure S3a, Supporting Information). We also note that during a prolonged 48 h‐long blebbistatin treatment, a subpopulation of MDA‐MB‐231 cells develops an extreme form of dendritic protrusions (Figure S3b, Supporting Information). However, despite a prolonged inhibition of actomyosin contractility with blebbistatin, the carcinoma cells maintain a high level of viability, i.e., ≈99% of the total population, as demonstrated by the detection of Caspase‐3/7 activity across the cell population (Figure S3c, Supporting Information).

To examine the contribution of the dynein motors to the protrusive elongation of cells, we use a selective dynein inhibitor dynarrestin (DA), which prevents dynein‐MT interactions^[^ ^33^ ^]^ and features a higher inhibiting efficiency compared to the ciliobrevin family.^[^ ^37^ ^]^ We refrain from using dynapyrazole A as a dynein inhibitor because, unlike DA, it blocks dynein's processive activity along the MTs, but does not prevent dynein‐MT interactions, rendering dyneins into the passive MT crosslinkers.^[^ ^38^ ^]^ Inhibition of dynein motors with dynarrestin in the presence of intact actomyosin contractility does not detectably affect MDA‐MB‐231 cell ability to adhere and protrude along the collagen grids (Figures S2a and 2a, Supporting Information). Cells preserve their polygonal morphology throughout the entire course of a 12‐hour‐long DA treatment, similar to the control cells (Figures S2a and 2a, Supporting Information). On the contrary, dynein suppression in the blebbistatin‐pretreated cells with low actomyosin contractility abruptly distorts the blebbistatin‐induced dendritic protrusions (Figures S2a and 2a, Supporting Information), followed by cells detachment from the collagen grids (Movie S5, Supporting Information). Thus, in the absence of substantial actomyosin contractility, dynein motricity becomes indispensable for maintaining cell protrusion, adhesion, and crawling.

Our results indicate that either myosin or dynein motors are able to provide some degree of cell protrusive spreading and crawling along the collagen grids while disabling both motor systems results in a complete loss of cell protrusion and adhesion, followed by detachment. Based on these observations, we suggest a model of cell locomotion powered by dynein‐generated forces within the microtubule network, which provides cell adhesion, formation of “dendritic”‐like cell protrusion, and migration (Figure 2b‐d). In this model, cells with decreased actomyosin contractility switch from the compact polygonal morphology to the extended “dendritic”‐like cell architectures (Figures 2b, S2a, and S3a, Supporting Information), using the mechanically loaded microtubule cables to transmit dynein‐generated forces along the “dendritic”‐like cell protrusions (Figure 2c,e).

The model of dynein‐generated motricity renders microtubules into the mechanically and structurally active component of the cytoskeleton,^[^ ^13^, ^14^, ^18^, ^39^ ^]^ which serves as a main bearing core of the “dendritic”‐like protrusions. Indeed, the seminal study of mesenchymal cell migration along the isolated one‐dimensional collagen lane and/or aligned collagen fibers phenomenologically highlighted the microtubules as a critical and indispensable component of cell protrusions that enables cell migration.^[^ ^28^ ^]^ We tested this hypothesis in our system by disassembly of the microtubules in the blebbistatin‐treated “dendritic” MDA‐MB‐231 cells on an 8.6 kPa collagen grid using 10 µM of nocodazole (Movie S6, Supporting Information). As expected, the disassembly of microtubules leads to disrupting the “dendritic”‐like protrusions, followed by their complete or partial disintegration into multiple bead‐like fragments (Figure S4a, Supporting Information).

The microtubule‐associated dynein motors are mechanically integrated into the F‐actin network via the dynactin complex, which is an F‐actin‐mimicking^[^ ^40^ ^]^ dynein cofactor.^[^ ^41^, ^42^, ^43^, ^44^ ^]^ Thus, dynactin enables a mechanical connection between the MT and F‐actin, as well as dynein‐driven MT sliding with respect to the F‐actin cytoskeleton. For example, the dynein's motricity has been tightly associated with both the displacement of individual microtubules^[^ ^45^ ^]^ and with the mechanically‐driven displacement and centering of the entire microtubular networks in reference to the F‐actin cytoskeleton spatial configuration.^[^ ^46^ ^]^ Mechanistically, dynactin may serve as a pseudo‐F‐actin filament, providing the nucleation seed for Arp2/3 complex assembly^[^ ^43^ ^]^ that, in turn, nucleates the branched F‐actin network around the microtubules. Therefore, dynactin can act as an adaptor between microtubules and cortical integrin‐based adhesion machinery, supporting the microtubules+dynein‐driven cell adhesion, microtubule‐based cell protrusion via “dendritic”‐like projections, and cell migration (Figure 2d,e). Thus, the dynactin‐dynein‐microtubule complex acts as a supporting system for the low‐contractility branched F‐actin network that grows around the microtubules in the dendritic protrusions. This suggestion is consistent with our previous report for MDA‐MB‐468 human breast cancer adenocarcinoma cell line, which, upon inhibition of Arp2/3 with CK666, shows the complete loss of the blebbistatin‐induced “dendritic”‐like protrusions, followed by the cell detachment,^[^ ^15^ ^]^ similar to cell detachment observed upon dynarrestin treatment in the blebbistatin‐pretreated MDA‐MB‐231 cells (Figures S2a and 2a, Supporting Information).

In summary, the proposed model for dynein‐driven migration of myosin‐attenuated cells outlines that the F‐actin‐integrated dynein motors slide and pull F‐actin towards microtubules’ (‐)‐ends and, in turn, pull the microtubules with respect to F‐actin towards their (+)‐ends, i.e., in the direction of the dendrites’ distal ends. Such dynein‐driven actin‐MT sliding generates the (+)‐end‐oriented protrusive forces, shown with red arrows in Figures 2c,d. The arising opposing reaction, i.e., mechanical tension forces within the F‐actin‐integrin‐collagen system are therefore directed towards the cell center as shown with black arrows in Figures 2c,d. Thus, the dynein‐driven MTs sliding towards the periphery of the cell along the collagen grid facilitates the network of dendritic protrusions and the extensive cell elongation, while the arising reaction forces on the substrate compensate for the diminished actomyosin tensile forces. A larger‐scale schematic model of the dynein‐driven cell protrusion and locomotion is presented in Figure 2e, where the dynein‐dynactin complexes, mechanically linked to the collagen via the F‐actin cortex and integrin‐based adhesion system, pull the MT cables in a “tug‐of‐war” manner against the reaction forces that constitute the tension on the substrate. The net effect of such force distribution is that dynein motors pull the entire cell toward the protruding end with respect to the substrate, creating directed motion. Indeed, dynein couples many organelles to the microtubules, but dynein‐mediated MTs‐nucleus^[^ ^47^, ^48^, ^49^, ^50^, ^51^ ^]^ coupling determines the entire cell body displacement, driven by the dynein motricity.

### 2.4 Genetic Attenuation of Dynactin‐Dynein Interactions Disables Dendritic Mode of Cell Protrusion

To challenge the proposed model of F‐actin‐to‐microtubules interlinking by the dynactin, we genetically manipulated MDA‐MB‐231 cells to target dynactin‐dynein complex assembly. Specifically, we over‐express the dynein‐binding CC1 domain (i.e., coiled coil‐1 domain) of the p150 (Glued) dynactin complex. According to the seminal study by Wu et al,^[^ ^46^ ^]^ the dynactin complex serves as a mechanical link between the microtubular dynein and the cell's cytomatrix. Over‐expression of the CC1 domain reportedly interferes with the dynein‐dynactin interactions via their competitive binding dynamics, resulting in disruption of the MT network linkage to the F‐actin/cytomatrix.^[^ ^46^ ^]^ In line with this report, the results of the human CC1‐GFP domain over‐expression in MDA‐MB‐231 cells display a suppression of the blebbistatin‐induced “dendritic”‐like protrusions in CC1‐GFP‐positive cells (Figure S4b, Movies S7, and S8, Supporting Information), accompanied by the overall shortening of all cell dimensions (Figure S4c, Supporting Information), compared to the CC1‐GFP‐low or ‐negative cells. These results indicate a substantial loss of the MT processivity along the cell adhesions and protrusions by the microtubule‐dynein‐dynactin‐F‐actin system,^[^ ^45^ ^]^ resulting in the failure of “dendritic”‐like mode of cell protrusion. Indeed, the morphometric analysis of protrusive dimensions in CC1‐GFP‐positive MDA‐MB‐231 cells on rigid collagen grids (*G*′ = 55 kPa) displays their average cell length at only ≈55 µm (Figure S4c‐1 and 4c‐2, Supporting Information) that is on par with ≈56 µm‐long cell elongation span for non‐transfected MDA‐MB‐231 cells on the same rigid collagen grids in control conditions (Figure S2c, Supporting Information).

### 2.5 Dynein is Indispensable for Microtubules Alignment to Nanotextured Adhesion Surfaces

In the context of the proposed model, we sought to evaluate the role of the dynein‐microtubules motricity in the mechanical reorganization and structural conformity of the microtubule network to the complex adhesion surfaces. Indeed, if the dynein‐driven spatial processing, sliding, and pulling of the microtubules occurs in respect to the F‐actin cortex, then the dynein motricity may have a direct effect on the microtubules conformity to the complex topography of F‐actin‐rich cell‐substrate adhesion interfaces. To test that, we utilize the plastic, collagen‐coated nano‐textures with 600 nm‐deep, 800 nm‐wide nano‐grooves spaced with 800 nm‐wide ridges to provide MDA‐MB‐231 cells with complex surface for adhesion and spreading for 12 h (Figure S5, Supporting Information). Results show that while MDA‐MB‐231 cells display a robust pulling and alignment of the microtubules into the underlying nano‐grooves in control conditions (Figures S5a and S5b‐1, Supporting Information), inhibition of dynein with dynarrestin results in a complete loss of the microtubules’ entrance and alignment within the underlying nano‐grooves (Figure S5a and S5b‐2, Supporting Information). Notably, suppressing the non‐muscle myosin II with blebbistatin does not preclude the microtubules from entering the nano‐grooves (Figure S5a, Supporting Information). Blebbistatin also does not prevent contact‐guidance‐driven alignment of MDA‐MB‐231 cells to nano‐textured surfaces, which is in line with the previously reported indifference of carcinoma cell‐nanotexture alignment to actomyosin contractility, dependent instead on microtubules‐nanotexture alignment.^[^ ^13^ ^]^ These results suggest that dynein processivity along microtubules is indeed required for the active entrance of the MT network into the underlying nano‐grooves within the cell‐nanotexture adhesion interface. The gathered observations are consistent with the proposed model of the dynein‐driven mechanical pulling and spatial reorganization of the microtubule network, which acts as a complementary system to conventional cell actomyosin contractility and spreading dynamics.

### 2.6 Microtubules, Dynein, Dynactin, and Adhesion Markers Colocalization in Dendritic Protrusions

We have also sought to capture the colocalization of the dynein and dynactin complexes with microtubules along with the integrin‐based adhesion‐associated markers in the highly “dentritized” blebbistatin‐treated MDA‐MB‐231 cells on collagen grids with 55 kPa stiffness (Figure S6, Supporting Information). The granular distribution of dynein complexes displays dynein colocalization with microtubules in the “dendritic”‐like protrusions (Figure S6a, Supporting Information). Similarly, the dynactin‐1 foci of dynactin complexes demonstrate colocalization with the microtubules within collagen‐guided “dendritic”‐like protrusions (Figure S6b, Supporting Information). The immature adhesion foci, labeled with the adhesion complex‐scaffolding paxillin protein,^[^ ^52^ ^]^ are also dispersed along the collagen‐guided dendritic protrusions (Figure S5c, Supporting Information). These results are consistent with previously established data for the breast carcinoma cells,^[^ ^13^, ^15^ ^]^ showing that the blebbistatin‐treated MDA‐MB‐231 cells do not develop large mature focal adhesions.

### 2.7 Simultaneous Inhibition of Dynein and Myosin Arrests Migration of Metastatic Cells

**Figure 3:**
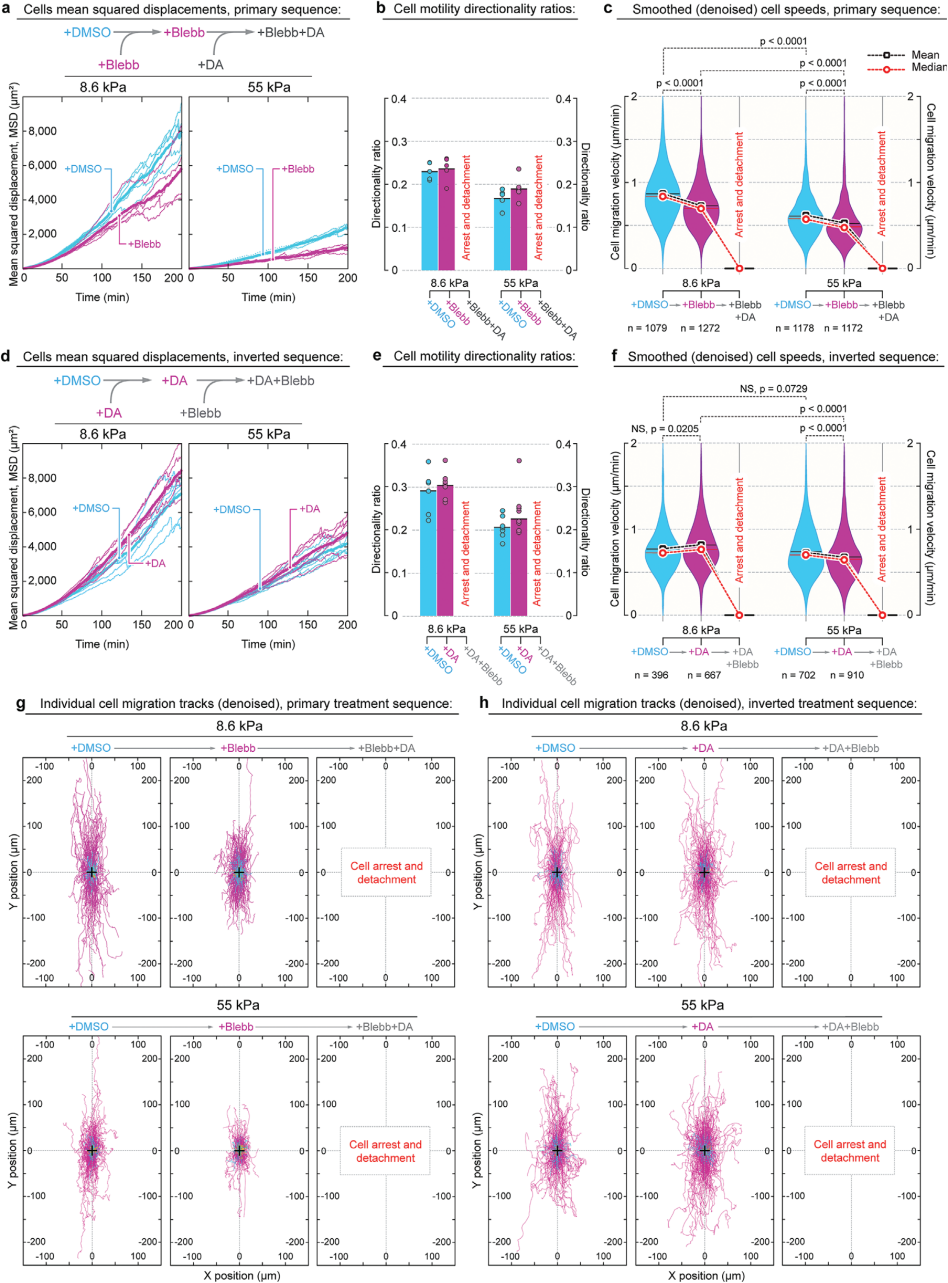
Metastatic MDA‐MB‐231 cells can utilize both myosin‐ and dynein‐driven systems to power collagen‐directed migration and contact guidance. a–c) Live cell migration imaging and analysis are performed sequentially (primary sequence) within the same cell samples and fields of view as follows: first – control conditions upon addition of the vehicle (+DMSO, cyan); second – during actomyosin contractility inhibition upon addition of blebbistatin (+Blebb, magenta); third – dynein suppression upon addition of dynarrestin over blebbistatin (+Blebb+DA, gray). Note that blebbistatin treatment does not arrest cell migration. Also note that MDA‐MB‐231 cells do not display any migratory properties (arrest and detachment) in the blebbistatin+dynarrestin mixture. a) Mean squared displacements (MSD) of MDA‐MB‐231 cells migrating along the soft (G′ = 8.6 kPa, left) and rigid (G′ = 55 kPa, right) collagen grids. b) Directionality ratios of the migrating MDA‐MB‐231 cells along the collagen grids. c) MDA‐MB‐231 cell migration speeds along smoothed (i.e., denoised) cell tracks for all treatment conditions. Note that suppression of actomyosin contractility with blebbistatin does not arrest but only marginally slows down migration of MDA‐MB‐231 cells. d–f) Sequential analysis of continuous cell migration across the following conditions (inverted sequence): first – control conditions (+DMSO, cyan); second – during dynein motor protein activity inhibition via addition of dynarrestin (+DA, magenta); third – actomyosin contractility suppression via addition of blebbistatin atop dynarrestin (+DA+Blebb, gray), i.e., +DMSO→+DA→+DA+Blebb sequence. d) Mean squared displacements of the MDA‐MB‐231 cells on soft (*G*′ = 8.6 kPa, left) and rigid (G′ = 55 kPa, right) collagen grids during the course of the sequential “inverted” treatments (i.e., +DMSO→+DA→+DA+Blebb). Note that dynein inhibition (+DA) does not significantly affect MDA‐MB‐231 cell displacement from the originating point compared to the same cells in control conditions (+DMSO). e) Directionality ratios of the migrating MDA‐MB‐231 cells along the collagen grids across all treatment conditions. f) MDA‐MB‐231 cell speeds along denoised trajectories for all treatment conditions. Note that dynein inhibition (+DA) does not significantly affect cell migration speeds compared to the same cells in control conditions (+DMSO). g) Individual smoothed (i.e., denoised) migration tracks of the MDA‐MB‐231 cells on soft (*G*′ = 8.6 kPa, top) and rigid (*G*′ = 55 kPa, bottom) collagen grids across +DMSO→+Blebb→+Blebb+DA conditions. h) Denoised migration tracks of the MDA‐MB‐231 cells on soft (*G*′ = 8.6 kPa, top) and rigid (*G*′ = 55 kPa, bottom) collagen grids across +DMSO→+DA→+DA+Blebb conditions. Note that in both +Blebb+DA and +DA+Blebb, MDA‐MB‐231 cells experience the migration arrest, followed by the cell detachment from soft and rigid grids.

We examine changes in MDA‐MB‐231 cell migration upon transition from the control conditions (+DMSO, ≈12 h) towards the conditions with experimental perturbations. For that, we first treated control cells with blebbistatin for 12 h and later added dynarrestin atop of the blebbistatin (**Figure** **3**a–c and Movie S9, Supporting Information). Alternatively, we used the inverted treatment sequence, i.e., treating cells with dynarrestin for 12 h followed by the addition of blebbistatin atop of dynarrestin (Figure 3d‐e and Movie S10, Supporting Information). Comparison of the cell responses between both sequences will allow us to separate and decipher the individual contributing roles of dynein and NM myosin II in cell motility.

In the first series of experiments, inhibition of actomyosin contractility only moderately decreases population‐wide effective migratory cell displacement, i.e., mean squared displacement (Figure 3a and Movie S9, Supporting Information). The population‐wide directionality ratio of migrating cells does not change significantly upon addition and incubation in blebbistatin (Figure 3b). Similarly, only a marginal decrease of the population‐wide cell speeds by Δ < 10% is observed during blebbistatin treatment on both soft (*G*′ = 8.6 kPa) and rigid (*G*′ = 55 kPa) collagen grids (Figure 3c). The consequent co‐inhibition of the dynein with dynarrestin, i.e., in addition to blebbistatin, leads to an abrupt arrest of cell motility (Figure 3c and Movie S5, Supporting Information), followed by the failure of the cell's dendritic protrusion and cell detachment from collagen grids (Figure 2a and Movie S5, Supporting Information).

In the second series of experiments, suppression of the dynein activity first with dynarrestin does not significantly affect MDA‐MB‐231 cell migratory behavior, compared to cells under the control conditions on soft and rigid collagen grids (Figure 3d‐f and Movie S10, Supporting Information). Specifically, inhibition of dynein activity on both soft (*G*′ = 8.6 kPa) and rigid (*G*′ = 55 kPa) collagen grids causes an insignificant increase of the population‐wide MSD (Figure 3d), cell motility directionality ratios (Figure 3e), and negligible change of the cell migration speeds (Figure 3f). The following co‐inhibition of the actomyosin contractility with blebbistatin atop the preceding ≈12‐h‐long dynein suppression (+DA) results in a cell arrest (Figure 3f).

Thus, our results show that the separate inhibition of either non‐muscle myosin II (+Blebb) or dynein (+DA) motors does not arrest the migration of MDA‐MB‐231 cells on soft (*G*′ = 8.6 kPa) and rigid (*G*′ = 55 kPa) collagen grids. However, simultaneous suppression of myosins and dyneins abruptly abrogates the migration of metastatic cells.

Although inhibition of actomyosin contractility (+Blebb) lowers the resulting mean squared displacement (MSD) of MDA‐MB‐231 cells, compared to isolated inhibition of dynein activity (+DA), MSD does not directly reflect the cell speed. Cell migration speed is measured as individual cell displacement along the migration track per unit of time, i.e., during both effective (directional) and ineffective (random‐walk‐like) cell movements. On the other hand, MSD is a surrogate metric that characterizes the contribution of cell movements toward their dispersion from the original position.

One of the principal contributing factors towards lowering of cell MSD upon +DMSO→+Blebb transition (Figure 3a) is the substantially longer span of blebbistatin‐treated cell protrusive spreading along the grids via “dendritic”‐like protrusions, compared to the cell in control conditions (Figure S2, Supporting Information). Thus, upon the addition of blebbistatin, each cell will occupy a substantially larger grid area, enhancing mutual cell‐cell crowding, which in turn will decrease the effective cell movements and cell MSD. On the other hand, dynarrestin treatment does not increase cell protrusion sizes (Figure S2, Supporting Information, +DMSO versus +DA), and therefore, does not significantly affect the cell‐cell crowding effects and effectiveness of cell movement.

**Figure 4:**
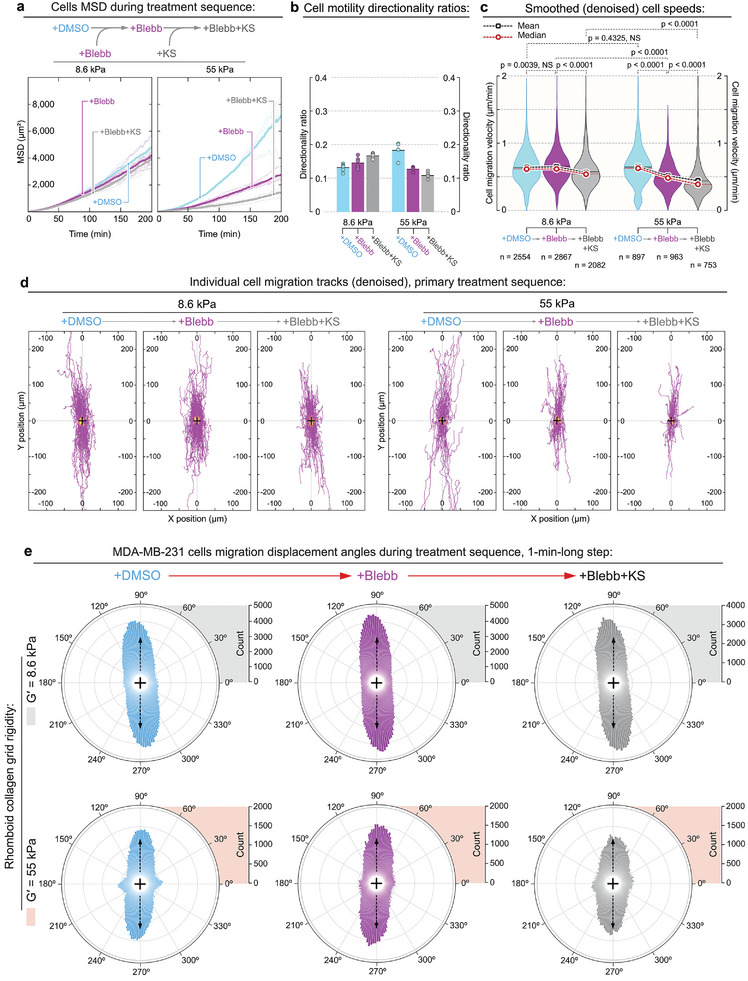
Orthogonal testing of the dynein‐driven metastatic locomotion of MDA‐MB‐231 cells with kinesore. Overactivation of dynein's mechanical antagonist, kinesin‐1, via treatment with kinesore (+KS) reduces cell locomotion but does not significantly affect the contact guidance along the collagen grid. Note that MDA‐MB‐231 cells do not lose migratory properties and do not detach during combined actomyosin contractility suppression and kinesin‐1 overactivation (+Blebb+KS) as dynein is not directly targeted and remains active. a) MSD of the MDA‐MB‐231 cells across a +DMSO→+Blebbistatin→+Blebbistatin+Kinesore treatment sequence reveals a substantial reduction of the cell displacement after addition of kinesin‐1 activator (+Blebb+KS) to the blebbistatin‐pretreated cells (+Blebb) on both soft (G′ = 8.6 kPa) and rigid (G′ = 55 kPa) collagen grids. b) Actomyosin contractility suppression (+Blebb) does not consistently change MDA‐MB‐231 cells directionality ratio in comparison to the control cells (+DMSO, see also Figure 2b, Supporting Information). Similarly, the addition of the kinesin‐1‐activating kinesore does not consistently change MDA‐MB‐231 ratio directionality ratio, see (+Blebb+KS). c) MDA‐MB‐231 cell migration speeds along denoised tracks under +DMSO→+Blebb→+Blebb+KS sequential treatment. Suppression of the actomyosin contractility with blebbistatin (+Blebb) moderately slows down MDA‐MB‐231 cell migration velocity. Overactivation of kinesin‐1 with kinesore atop actomyosin contractility suppression by blebbistatin (+Blebb+KS) further slows cell migration speeds, indicating a possible mechanical antagonism between kinesin‐1 and dynein mechanical forces during the suppression of actomyosin contractile forces. d) Individual denoised migration tracks for the MDA‐MB‐231 cells on soft (G′ = 8.6 kPa, top) and rigid (*G*′ = 55 kPa, bottom) collagen grids across +DMSO→+Blebb→+Blebb+KS treatment sequence. e) MDA‐MB‐231 cell migration direction distributions in control (+DMSO, left), during actomyosin contractility inhibition (+Blebb, center), followed by the combined kinesin‐1 activation atop actomyosin contractility suppression (+Blebb+KS, right), on both soft (*G*′ = 8.6 kPa) and rigid (*G*′ = 55 kPa) collagen grids. Displacement directions are compiled into circular diagrams with the grid anisotropy axis oriented vertically (arrows).

In fact, our data shows that none of the single treatments with either blebbistatin or dynarrestin significantly affects MDA‐MB‐231 cell locomotion speeds. For example, blebbistatin treatment induces only a marginal (Figures 3c, +DMSO→+Blebb), or even a statistically insignificant slowdown of cell speeds (**Figures** **4**c, +DMSO→+Blebb). Similarly, treatment of MDA‐MB‐231 cells with dynarrestin also induces the marginal, sometimes statistically insignificant changes in cell speeds (Figure 3f, +DMSO→+DA). Only the combined +Blebb+DA or +DA+Blebb treatments invariably ensure a complete cell arrest or even cell adhesion failure and detachment from the substrate (Figure 3c,f, +Blebb+DA and +DA+Blebb). Thus, perhaps the most important observation is the consistently reproducible decrease of the MSD during +DMSO→+Blebb transition for each individual experiment (Figures 3a and 4a), albeit to a variable extent. Such consistent MSD decrease reflects the MDA‐MB‐231 cells’ transition towards “dendritic”, much longer span of cell protrusions, causing the enhanced cell‐cell crowding and collisions, and decreased efficiency of cells’ long‐term dispersion (not necessarily decreasing mean cell speed along their individual tracks).

Importantly, we also note that the other invariable observation is the consistently reproducible decrease of the MSD during +DMSO→+Blebb transition for each individual experiment (Figures 3a and 4a), albeit to a variable extent. Indeed, the consistent decrease of MSD reflects the MDA‐MB‐231 cells transition towards “dendritic”‐like, much longer span of cell protrusions, causing the enhanced cell‐cell crowding and decreased efficiency of cells’ long‐term dispersion, but not necessarily the decreased mean cell speed along their individual tracks.

These results indicate that cell motility is a complex process, where dynein‐ and myosin‐driven locomotion mechanisms compensate for each other with great efficiency, but their interplay is a multiparametric process that introduces a substantial variability into the cell speeds and displacements between the individual experiments. Specifically, the cell population density may have an insignificant impact on the control MDA‐MB‐231 cells, but its impact increases upon the blebbistatin‐induced enhancement of cell lengths and frequency of cell‐cell collisions.

### 2.8 Isolated Myosin‐ or Dynein‐Driven Locomotion Supports Contact Guidance of Metastatic Cells

To analyze the directional responsiveness of MDA‐MB‐231 cancer cells to the geometry of the anisotropically oriented collagen grid, i.e., contact guidance,^[^ ^13^, ^15^, ^53^, ^54^ ^]^ we evaluate individual tracks of migrating cells (Figure 3g,h) and the total angle distribution of 1‐min‐long cell displacement in respect to the grid's anisotropy axis (Figure S7, Supporting Information) for all used conditions.

Our results indicate that the directionality of cell migration is equally responsive to the anisotropy of the collagen contact guidance cues (grids) on soft (*G*′ = 8.6 kPa) and rigid (*G*′ = 55 kPa) substrates in control conditions (Figure 3g,h, and Movies S9 and S10, Supporting Information), during inhibition of the actomyosin contractility (Figure 3g and Movies S9 and S10, Supporting Information), or during the suppression of the dynein (Figure 3h and Movies S9 and S10, Supporting Information).

Similarly, the angle distributions of cell displacements in relation to the grid anisotropy show no significant deflection of the cell migration course from the directionality of the rhomboid grid anisotropy axis of both soft (*G*′ = 8.6 kPa) and rigid (*G*′ = 55 kPa) substrates for all treatment conditions (Figure S7a,b, Supporting Information). Thus, our data demonstrate that migrating MDA‐MB‐231 cells can sustain contact guidance via either myosin‐ or dynein‐driven mechanism or both.

### 2.9 Upregulation of Kinesin‐1 Activity Attenuates Dynein‐Driven Locomotion

To further challenge the model of dynein‐driven cell motility during diminished actomyosin contractility, we utilize the previously reported^[^ ^55^ ^]^ mechanical antagonism between dynein and kinesin‐1 in carcinoma cells. Specifically, the reported study describes mechanical antagonism between kinesin‐1 and dynein in MDA‐MB‐231 cells that determines their microtubules architecture, cell contact guidance, and microtubules alignment to the guidance cues, which can be utilized as an orthogonal test to the inhibition of the dynein in cells with suppressed actomyosin contractility (Figures 2 and 3). For that purpose, we study the migratory response of blebbistatin‐pretreated cancer cells to the kinesin‐1 overactivation induced by a small kinesin‐1‐specific activator, kinesore (KS).^[^ ^14^, ^56^ ^]^

The addition of kinesore to the blebbistatin‐pretreated MDA‐MB‐231 cells (Figure 4a‐c) results in a substantial slow‐down of cell locomotion on both soft (*G*′ = 8.6 kPa) and rigid (*G*′ = 55 kPa) collagen grids (Figure 4a‐c, Movie S11, Supporting Information). Thus, over‐stimulation of kinesin‐1 activity and its mechanical antagonism with dyneins^[^ ^14^ ^]^ may indeed interfere with the dynein‐driven cell locomotion. Moreover, since kinesore does not inhibit dynein but only over‐activates kinesin‐1, we do not observe detachment of cells, as opposed to dynarrestin addition to the blebbistatin‐pretreated MDA‐MB‐231 cells (Figures 2a and 3c).

Similarly, we examine kinesore‐induced changes to contact guidance of cells moving on the rhomboid collagen grid (Figure 4d‐e). The quantification of contact guidance shows no significant loss of cell directionality, as all MDA‐MB‐231 cells continue to predominantly migrate along the main grid axis on both soft (*G*′ = 8.6 kPa) and stiff (*G*′ = 55 kPa) substrates, as shown by individual cell migration tracks (Figure 4d) and the angular distribution of 1‐min‐long displacements from anisotropy axis of the grid (Figure 4e).

Examination of the kinesin‐1 overactivation on the microtubules’ ability to enter into the nano‐grooves as MDA‐MB‐231 cells spread on the collagen‐coated nano‐textures displays the failure of microtubules to interact with and align to the presented topographic features (Figure S5a, Supporting Information). These observations may point towards the active retraction of the microtubules from the nano‐grooves via counterbalancing the dynein's processivity.^[^ ^55^ ^]^ Kinesin‐1 actively crosslinks microtubules to each other,^[^ ^57^, ^58^, ^59^ ^]^ and disproportionate activity of kinesin‐1 drives antiparallel microtubule‐microtubule sliding into the MT bundles or cortical rings in the direction away from the cell adhesion sites.^[^ ^55^ ^]^ Notably, kinesore‐induced loss of the in‐groove microtubules causes instead a previously reported microtubules reassembly into the cell‐expanding rings that visibly reorganize cells into the circular morphology (Figure S5a, Supporting Information).^[^ ^14^ ^]^ Loss of the in‐groove microtubules suppresses cell contact guidance‐like spreading and alignment of the MDA‐MB‐231 cells to the underlying nano‐textures, a phenomenon that has also been previously strongly attributed to the microtubules’ ability to sterically interact with the nano‐texture.^[^ ^13^ ^]^

All listed above findings indicate that although kinesore‐induced overactivation of kinesin‐1 does not preclude dynein‐driven cell adhesion, “dendritic”‐like protrusive spreading, and contact guidance, it partially, yet substantially attenuates the motility speed of cancer cells. However, the proposed dynein‐kinesin‐1 mechanical antagonism warrants further, more detailed investigations to determine the extent of the described mechanical competition between the two motor systems,^[^ ^14^ ^]^ and the degree of its contribution to the cancer cell locomotion, a substantial task that remains beyond the scope of this study. Thus, together with the results from the previous experiments using direct dynein inhibition, kinesin‐1 activation data is consistent with the conception that dynein motricity complements actomyosin contractility, providing locomotion and contact guidance in metastatic MDA‐MB‐231 cells.

### 2.10 Adhesion and Protrusion of Nonmetastatic Cancer Cells Feature Limited Dependence on Dynein

**Figure 5:**
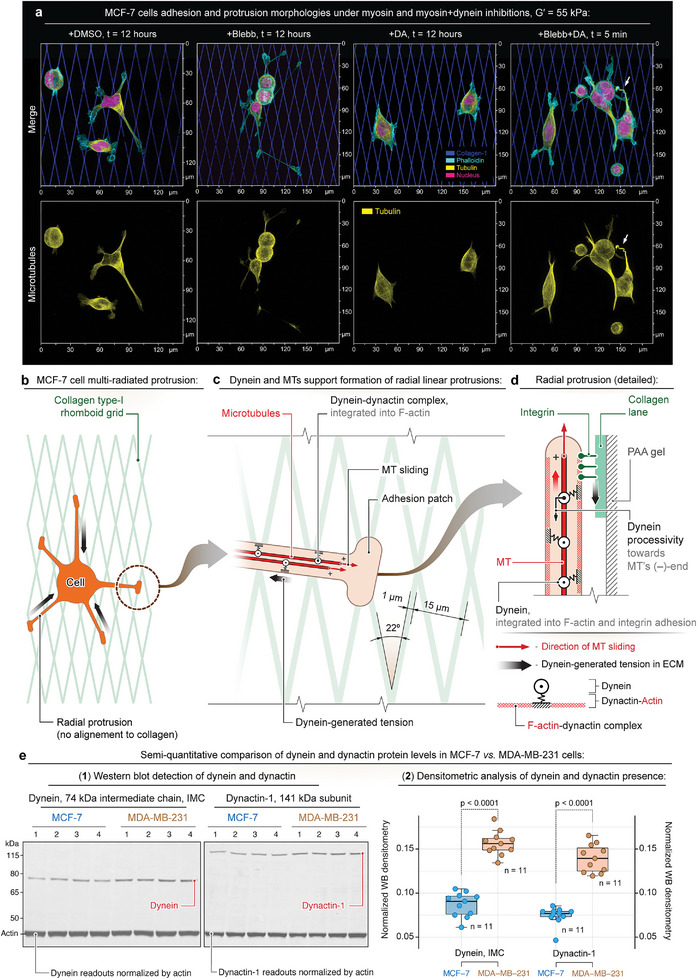
Adhesion and protrusion of non‐metastatic MCF‐7 breast cancer cells on collagen grids display limited dependence on dynein‐generated tension and diminished contact guidance. a) Adhesion and protrusion of MCF‐7 cells on stiff (*G*′ = 55 kPa) collagen grids in control conditions (+DMSO), upon actomyosin contractility inhibition with blebbistatin (+Blebb), upon dynein inhibition with dynarrestin (+DA), and after consequential addition of blebbistatin and dynarrestin (+Blebb+DA). Note that, unlike MDA‐MB‐231, the MCF‐7 cells form radial (non‐dendritic) cell protrusions with limited adhesion restricted to their tips. Limited adhesion prevents adhesive alignment and conformity of MCF‐7 protrusions to the collagen grid (+DMSO). Blebbistatin further reduces cell structural coherence by enhancing the length and number of independent radial cell protrusions (+Blebb). Note that inhibition of dynein abrogates linear protrusions, resulting in simplified, polygonal MCF‐7 cell architecture (+DA). Combined inhibition of actomyosin and dynein contractilities leads to complete cell detachment from soft (*G*′ = 8.6 kPa, Figure S8a, Supporting Information) and to partial detachment from rigid (*G*′ = 55 kPa) collagen‐1 grids with gradual failure of the radial protrusions (arrow). b) Schematic view of the MCF‐7 cell with multiple radial protrusions in controlconditions (+DMSO) that do not facilitate cell alignment to the collagen grid anisotropy. c,d) Suggested schematic representation of the radial protrusions‐collagen grid interactions. The dynein motricity slides the MTs towards the tip of radial protrusion (red arrows), while the induced reaction to the dynein‐F‐actin displacement along the MT within the radial protrusions generates the tension in the underlying ECM (black arrows). Note that the dynein‐based structural integration of microtubules into the radial cell protrusions provides long‐distance force transduction and tension, which stimulate the adhesion of distal radial protrusions’ tips to the collagen grid, but not the adhesive alignment and conformity of protrusions to the collagen grid along their entire lengths. e) Semi‐quantitative analysis of protein levels for dynein's cytosolic intermediate chain (i.e., dynein IMC, antibody clone 74.1) and dynactin‐1 (antibody clone 3D5‐C6‐D5) in MCF‐7 and MDA‐MB‐231 cell lysates by western blot. (1) Examples of raw images, with each lane displaying a 10‐µg‐load of MCF‐7 or MDA‐MB‐231 cell lysate, prepared independently. The total number of independently prepared and analyzed lysates is n = 11 for each cell line. (2) Densitometric analysis of western blots. For analysis of dynein and dynactin‐1 levels, their intensity values were normalized using corresponding intensity values of β‐actin loading control. Densitometric analysis shows a 77 percent higher level of dynein and a 75% higher level of dynactin‐1 protein in MDA‐MB‐231 cells compared to the MCF‐7 line. Data are shown as box and whisker diagrams: first quartile, median, third quartile, and 95% percent confidence interval.

The metastatically aggressive^[^ ^60^ ^]^ and fully EMT‐transitioned^[^ ^61^ ^]^ triple‐negative MDA‐MB‐231 breast cancer cell line is one of the characterized models for metastasis studies.^[^ ^9^, ^53^, ^62^ ^]^ To further study the specific role of dynein, microtubules, and dynactin in metastasis, we choose a commonly used model of non‐metastatic breast cancer cells^[^ ^63^ ^]^ – a hormone receptor‐positive noninvasive MCF‐7 cell line, which features an incomplete epithelial‐mesenchymal transition (EMT).^[^ ^64^ ^]^ Presenting control MCF‐7 cells to the collagen grids results in the formation of the radial (i.e., non‐’dendritic’‐like) protrusions that display limited adhesion restricted to the radial protrusions’ tips in control cells on both soft (Figure S8a, Supporting Information) and rigid substrates (**Figure** **5**a, Movie S12, Supporting Information). Limited adhesion of MCF‐7 cells prevents their congruent protrusion alignment to the collagen grids (Figure 5b). Inhibition of the dynein activity with dynarrestin suppresses the formation of radial protrusions, inducing polygonal morphology of MCF‐7 cells on both soft and rigid collagen grids (Figures S8a, Supporting Information, and 5a).

Suppression of the actomyosin contractility with blebbistatin enhances the formation of radial protrusions, reducing cells’ structural coherence, but it does not increase the structural alignment of the protrusions to soft and rigid collagen grids (Figures S8a, Supporting Information, and 5a). This observation indicates that similarly to MDA‐MB‐231 cells, the dynein in MCF‐7 cells may also play a key role in the protrusion adhesions. Indeed, suppression of dynein activity in blebbistatin‐treated MCF‐7 cells results in the retraction of radial protrusions, cell arrest, and detachment from the soft collagen grids (Figure S8a, Supporting Information). Similarly, suppressing dynein activity in blebbistatin‐treated MCF‐7 cells on the rigid collagen grids leads to a significant slow‐down and partial detachment of the radial protrusions (Figure 5a). These results are consistent with MDA‐MB‐231 data but demonstrate a more limited role of dynein motricity in providing the structural conformity to the underlying collagen grids.c

Insufficient dynein motricity in MCF‐7 cells may contribute to the lack of congruent alignment of cell protrusions to the collagen grids, compared to MDA‐MB‐231 cells (Figure 2c,e). However, dynein motricity in MCF‐7 cells is still required to maintain the formation of the radial cell protrusions on collagen grids (Figure 5c). Indeed, multiple reports indicate the importance of the dynein activity^[^ ^16^, ^17^ ^]^ and intact microtubules^[^ ^13^, ^14^, ^15^, ^65^ ^]^ for alignment and conformational branching of cell protrusions along the adhesion cues. Therefore, we suggest that the dynein‐dynactin‐microtubules system generates the motricity within the radial protrusions in MCF‐7 cells (Figure 5b‐d) sufficient only for mechano‐stimulatory tensile forces to activate integrin adhesion via the mechanosensory mechanisms at the tips of the radial protrusions.^[^ ^66^ ^]^

The morphological analysis of the MCF‐7 cells across all conditions demonstrates mechanosensitive responses of cells to underlying substrates and treatments. Specifically, MCF‐7 cells display an increased formation of radial protrusion and polygonal spreading on the rigid grids, compared to the soft grids (Figure S8b,c, Supporting Information). Such differential cell response to the adhesive grid rigidity highlights an elevated mechanosensitivity of MCF‐7 compared to the MDA‐MB‐231 cells (Figure S2c, Supporting Information). For MCF‐7 cells, adhesions are visibly limited to the tips of the radial protrusions, attached to the collagen lanes. The converged and accumulated tension at the radial protrusions’ tips may become critical for modulating the integrin mechanosensitive response and adhesion. On the contrary, spatially distributed tension along the continuous adhesion within the “dendritic”‐like protrusions of MDA‐MB‐231 cells, which congruently conform and adhere to the collagen grids throughout their entire lengths, likely allows for the effective microtubules sliding and protrusion with the lower tension generated by each individual dynein molecule. Thus, while the high degree of spatial distribution of the adhesion in MDA‐MB‐231 cells along their “dendritic”‐like protrusions renders them less dependent on the rigidity of the adhesion substrate, the few and spatially limited adhesions in MCF‐7 cell render them more dependent on the rigidity of the adhesive substrate.

In addition to the protrusion tip‐limited adhesions, the insufficient dynein motricity in non‐metastatic MCF‐7 cells may be linked to a lower expression of dynein and its cofactor dynactin, compared to metastatic MDA‐MB‐231 cells (RNA expression data are available via The Human Protein Atlas database).^[^ ^25^ ^]^ The semi‐quantitative western blot analysis of the cytoplasmic dynein intermediate chain and dynactin‐1 subunit across eleven independently prepared lysates of each MCF‐7 and MDA‐MB‐231 cell lines, consistently indicates substantially higher protein level (≈75%) for both dynein and dynactin in metastatically aggressive MDA‐MB‐231 cells, compared to nonmetastatic MCF‐7 cells (Figure 5e). Similarly, the flow cytometry single‐cell analysis of the immuno‐fluorescently labeled intracellular dynactin‐1 indicates an average ∼90% margin in MDA‐MB‐231 cells over MCF‐7 cells (Figure S9, Supporting Information).

### 2.11 Limited Dynein Motricity Diminishes Contact Guidance of Nonmetastatic Cells

**Figure 6:**
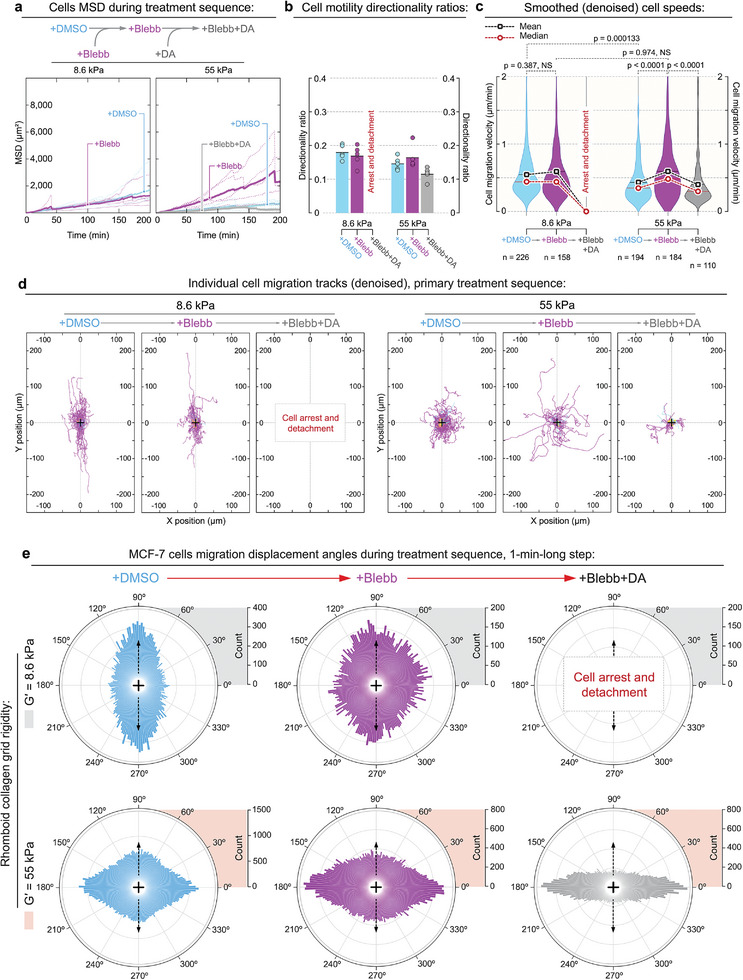
Non‐metastatic MCF‐7 breast cancer cells display a limited dependence on dynein activity for their adhesion and alignment to the 2D collagen contact guidance cues. a) MSD of the MCF‐7 cells across +DMSO→+Blebbistatin→+Blebbistatin+Dynarrestin treatment sequence displays an increase of cell displacement after addition of non‐muscle myosin IIA inhibitor blebbistatin (+Blebb) on rigid (G′ = 55 kPa) collagen grids. Note that on soft (*G*′ = 8.6 kPa) collagen grids, the blebbistatin‐treated MCF‐7 cells arrest and detach upon dynein inhibition while also aggregating into the clusters via E‐cadherin cell‐cell interactions. Also note that on rigid (*G*′ = 55 kPa) collagen grids, cells display a partial detachment and reduce their migration speeds. b) MCF‐7 cells do not display a strong fluctuation of their directionality ratios (persistence) across all conditions and rigidities. c) MCF‐7 migration speeds along denoised tracks under +DMSO→+Blebb→+Blebb+DA sequential treatment. Suppression of the actomyosin contractility with blebbistatin (+Blebb) moderately enhances MCF‐7 motility on both soft (*G*′ = 8.6 kPa) and rigid (*G*′ = 55 kPa) collagen grids, indicating blebbistatin‐induced enhancement of the mesenchymal‐like polarization of the cell with an incomplete EMT transition. Further inhibition of the dyneins, in addition to suppression of actomyosin contractility, results in the detachment of MCF‐7 cells from the soft collagen grids (*G*′ = 8.6 kPa) while inducing a decrease of both cell speeds and directionality ratio on rigid grids (*G*′ = 55 kPa), leading to a significant decrease of cells’ mean squared displacement, see panel (a). d) MCF‐7 cells’ denoised migration tracks on soft (*G*′ = 8.6 kPa, top) and rigid (*G*’ = 55 kPa, bottom) collagen grids for +DMSO→+Blebb→+Blebb+DA treatment sequence. Note the complete loss of cell migration track alignment to the stiff collagen grid (*G*′ = 55 kPa). e) MCF‐7 cell migration direction distributions in control (+DMSO, left), during actomyosin contractility inhibition (+Blebb, center), and during dynein co‐suppression (+Blebb+DA, right), on both soft (*G*′ = 8.6 kPa) and rigid (*G*′ = 55 kPa) collagen grids. Displacement directions are compiled into circular diagrams with the grid anisotropy axis oriented vertically (arrows). Note that, unlike metastatic MDA‐MB‐231 breast cancer cells, the non‐metastatic MCF‐7 breast cancer cells on the rigid collagen grids (*G*′ = 55 kPa) display opposite response to the contact guidance cues, i.e., predominantly displays transverse cell displacements with respect to the axis of underlying contact guidance cues. Note that most of the cell 1‐min‐long displacements do not lead to effective transverse migration across the collagen grid (see panel (d), right), indicating a low efficiency of the transverse forces.

Compared to the control MDA‐MB‐231 cells (Figure 3a‐c), the control MCF‐7 cells show lower cells’ mean squared displacements and slower locomotion under control conditions (**Figure** **6**a–c). We also observe a moderate acceleration of MCF‐7 cell speeds during inhibition of the actomyosin contractility (Figure 6a‐c), perhaps, due to the previously described effects of blebbistatin‐induced symmetry breaking and cell transitioning from epithelial‐like apical‐basal polarity to the migration‐effective mesenchymal‐like front‐back polarity.^[^ ^67^ ^]^ Thus, in our experiments, blebbistatin treatment is accompanied by the enhancement of motility on both soft (*G*′ = 8.6 kPa) and rigid (*G*′ = 55 kPa) collagen grids (Figure 6c), compared to the control cells.

Since epithelial‐like MCF‐7 cells feature an incomplete EMT, the underdeveloped mesenchymal organization generates insufficient actomyosin‐driven ECM traction for cell migration.^[^ ^68^, ^69^ ^]^ The lack of mesenchymal‐like actomyosin contractility within the cell‐ECM adhesion interface increases the dependence of MCF‐7 cells on the scarce dynein‐microtubule motricity within the radial protrusions (Figure 5c‐d), as confirmed by the loss of radial protrusions upon inhibition of dynein activity (Figures S8 and 5a, Supporting Information). Similarly, the role of dyneins in the attachment of MCF‐7 cells to the substrate is demonstrated by the complete and partial detachment of cells upon inhibition of dyneins in blebbistatin‐treated MCF‐7 cells on soft and rigid collagen grids, respectively (Figures 6c S5, and 5a, Supporting Information).

As opposed to the “dendritic”‐like protrusions in MDA‐MB‐231 cells (Figures S2 and 2a, Supporting Information), the radial protrusions in MCF‐7 cells do not support persistent contact guidance along the collagen grids. Yet, relative to the anisotropy axis of the collagen grid (Figure 6e), the individual migration tracks of cells on soft collagen grids show moderate conformity of MCF‐7 cells migration along the collagen cues in control conditions and during actomyosin inhibition (Figure 6d). However, the angular distribution of cells’ 1‐min‐long displacement on the soft collagen grids demonstrates a significant disarray (Figure 6e), exaggerated on the rigid collagen grids (Figure 6e). Specifically, we observe a predominantly transverse orientation of MCF‐7 cells’ movements with respect to the collagen grids' anisotropy axis on rigid grids (Figure 6e). The transverse displacement without a significant change in the final cell migration track along the soft collagen grid points towards the radial protrusion‐driven oscillation of MCF‐7 cells across the grids. These oscillations do not result in effective cell displacement, but indicate a growing role of radial protrusions in cell‐collagen interactions that become substantially more evident in MCF‐7 cells migrating along the rigid collagen grids (Movie S12, Supporting Information).

In particular, at the level of the individual cell migration tracks, we observe a complete loss of migration conformity in MCF‐7 cells to the rigid (*G*′ = 55 kPa) collagen grid in all conditions (Figure 6d). We detect more frequent transversely oriented 1‐min‐long displacements in relation to the collagen grid anisotropy axis (Figure 6e), which become a dominant orientation for MCF‐7 cells in all conditions. Similarly to results on the soft collagen grids, the majority of transverse displacements are not effective, with resulting cell migration tracks displaying a random migration in relation to the underlying rigid collagen grids (Figure 6d). These observations indicate that cell contact guidance requires, at least in part, an alignment of cell protrusions to the underlying collagen guidance cues. This alignment is facilitated by the alignment of the microtubules in the protrusions via the motricity of dynein‐dynactin‐microtubules complexes and their mechanical coupling with F‐actin. These observations are consistent with previous reports showing the crucial role of the microtubule alignment to the substrate cues during cell contact guidance.^[^ ^13^, ^55^ ^]^

### 2.12 Confined 3D Migration Requires the Combined Activity of Dynein and Myosin Motors

The hallmark of metastasis is the ability of cancer cells to migrate in the confinement of tissues. To test the role of myosin and dynein motors in the confined 3D migration, we investigate the effects of dynarrestin and blebbistatin on MDA‐MB‐231 motility in granular hydrogel scaffolds (GHS). Herein, we fabricate GHS using gelatin methacryloyl (GelMA) microgel building blocks with different GelMA concentrations and varying light exposure times to provide a range of mechanical stiffnesses (Figure S10, Supporting Information).

**Figure 7:**
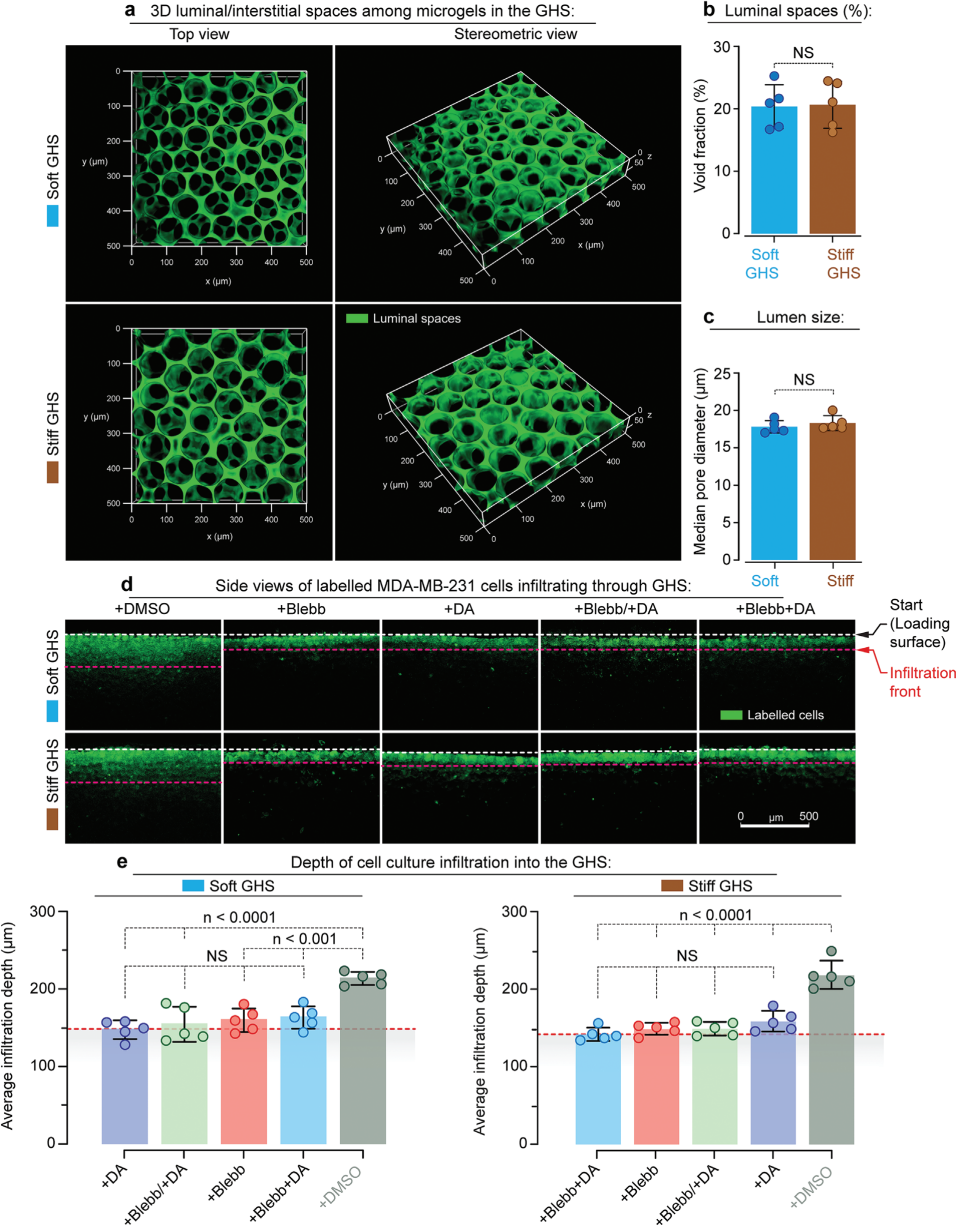
Migration of metastatic MDA‐MB‐231 breast cancer cells in GHS depends on the simultaneous activity of dynein and myosin motors. a) 3D microscopy images of GHS made up of soft or rigid GelMA microgel building blocks. The void space is occupied by fluorescently conjugated high molecular weight dextran molecules that do not penetrate the microgels. b) Void fraction and c) median pore diameter of soft and rigid GHS, showing no significant difference between them. d) Sagittal view of sectioned scaffolds 72 h after topical cell seeding, with white and red dashed lines indicating the uppermost layer of GHS and the average cell migration depth, respectively. Cells treated with blebbistatin (+Blebb), dynarrestin (+DA), Blebb starting at *t* = 0 h, followed by DA starting at *t* = 12 h (+Blebb/+DA), and both Blebb and DA starting at *t* = 0 h (+Blebb+DA), showed limited motility compared with untreated cells in the control group (scale bar is 500 µm). e) Average infiltration depth of cells treated with the inhibitors of cell contractility/tension in GHS (*n* = 5). Dashed lines represent the average initial cell infiltration length (≈4 h after topical cell seeding) in soft (147 ± 9 µm) and rigid (138 ± 6 µm) GHS, wherein no statistically significant difference was observed compared with all the treated groups.

Then, we characterize the porosity of soft (*G*′ ≈ 8.9 ± 0.6 kPa) and rigid (G′ ≈ 52.2 ± 4.8 kPa) GHS using fluorescence microscopy of a high molecular weight fluorescent dextran solution (**Figure** **7**a, Movies S13 and S14, Supporting Information). The void fraction (Figure 7b), pore identification (Figure S11, Supporting Information), and corresponding median pore diameter (Figure 7c) show a nonsignificant difference between the void fraction for soft (20 ± 4%) and rigid (21 ± 4%) GHS, as well as for median pore diameter: 17.8 ± 0.8 µm and 18.3 ± 1.0 µm for the soft and rigid GHS, respectively.

**Figure 8:**
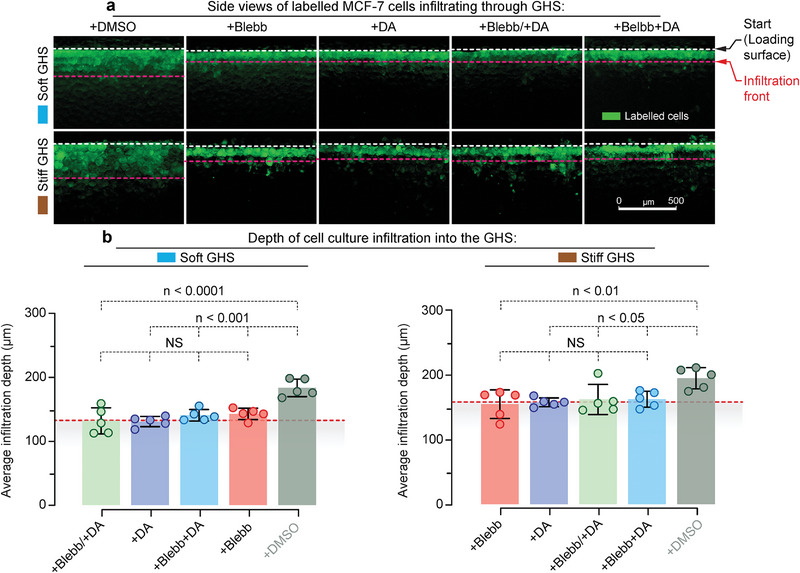
Migration of non‐metastatic MCF‐7 breast cancer cells in GHS depends on the simultaneous activity of dynein and myosin motors. a) Sectioned side view of the MCF‐7 cells‐infiltrated scaffolds 72 h after topical cell seeding, with white and red dashed lines indicating the uppermost layer of GHS and the average cell migration depth, respectively. Cells treated with blebbistatin (+Blebb), dynarrestin (+DA), Blebb starting at *t* = 0 h, followed by DA starting at *t* = 12 h (+Blebb/+DA), and both Blebb and DA starting at *t* = 0 h (+Blebb+DA), showed limited motility compared with untreated cells in the control group (scale bar is 500 µm). b) Average infiltration depth of cells treated with the inhibitors of cell contractility/tension in GHS (*n* = 5). Dashed lines represent the average initial cell infiltration length (≈4 h after topical cell seeding) in soft (132 ± 14 µm) and stiff (160 ± 10 µm) GHS, wherein no statistically significant difference was observed compared with all the treated groups.

To study the confined 3D migration of metastatic MDA‐MB‐231 cells, we seed fluorescently labeled cells on top of the GHS and analyze their migration after 72 h. The cross‐sectional microscopy of cell‐seeded scaffolds shows no difference between control cells within soft and rigid GHS substrates (Figure 7d,e). However, the confined 3D migration of blebbistatin‐, dynarrestin‐, and blebbistatin+dynarrestin‐treated MDA‐MB‐231 cells is lower (Figure 7d,e) compared to control cells. Moreover, the average migration length of all treated samples is limited to the extent of initial cell penetration, measured ≈4 h after seeding, where gravitational and capillary forces mainly govern cell entrainment. We observe similar results for non‐metastatic MCF‐7 breast cancer cells (**Figure** **8**a–b). These data indicate that confined 3D migration has more stringent requirements for the simultaneous activity of myosin and dynein motors.

## 3 Discussion

Since mesenchymal cancer cells often migrate and exit the primary tumor throughout and along the fibrous collagen‐rich extracellular matrices, the biomimetic substitutes of ECM, such as 3D collagen gels, became major models for metastasis studies.^[^ ^9^, ^15^, ^28^, ^29^, ^53^, ^62^, ^70^, ^71^, ^72^ ^]^ Nevertheless, the structural and mechanical complexity of 3D collagen gels effectively prohibits detailed mechanobiological studies of the motile behavior of cancer cells in ECM.^[^ ^73^, ^74^ ^]^ A question remains whether actomyosin‐driven adhesion and contractility alone are sufficient to coordinate, integrate, and provide a coherent^[^ ^67^, ^75^ ^]^ and persistent^[^ ^76^ ^]^ cancer cell migration within structurally anisotropic and noisy 3D collagen matrices.^[^ ^77^ ^]^

Therefore, to examine the role of actomyosin and microtubule motors during metastasis, we study MDA‐MB‐231 cells’ migratory behavior on the soft (shear modulus *G*′ = 8.6 kPa) and rigid (*G*′ = 55 kPa)^[^ ^31^, ^32^ ^]^ 2D biomimetic substrates, which consist of crisscrossed 1‐µm‐wide parallel collagen type‐1 lanes, i.e., artificial collagen “fibers” that mimic naturally occurring collagen architectures.^[^ ^2^, ^53^, ^78^ ^]^ We chose the pitching distance between each set of the parallel collagen lane at 15 µm, as it provides a sufficient spatial sparsity of the adhesion cues to prevent the loss of the cell's sensitivity to the guidance effects of the collagen architecture.^[^ ^13^, ^15^, ^79^ ^]^ The rationale for the chosen collagen lanes’ width and spacing between them is also based on the direct observations of the breast and pancreatic carcinoma metastasis‐promoting tumor‐associated collagen signatures (TACS‐3), where the micron‐scale^[^ ^80^ ^]^ collagen fibers re‐orient perpendicularly to the boundaries of the tumor,^[^ ^2^ ^]^ promoting carcinoma cells metastatic escape.^[^ ^10^ ^]^ In these microenvironments, the collagen topography and architecture feature single or grouped collagen fibrils, spaced in a multiscale manner with a range of hundreds of nanometres to a few micrometers apart, with a mean spacing of ≈850 nm between fibril bundles.^[^ ^2^, ^80^ ^]^ Moreover, a cell or cell collective migration along the 3D collagen matrix often pulls and bundles the dense multiple submicron collagen fibers into the larger micron‐scale fibrils, rendering them into the spatially separated and pronounced anisotropic guidance cues, resembling the utilized collagen rhomboid grids^[^ ^81^, ^82^ ^]^ Thus, the key characteristics of the collagen matrix surrounding the metastatically aggressive carcinoma tumors are its structural, spatially anisotropic architecture and multiscale organization of their fibril density. We aimed to mimic such structural organization of the collagen architectures while also controlling the mechanical rigidity of the collagen guidance cues in the setting, compatible with the high throughput live cell imaging microscopy methods. To achieve this goal, we chose the reductionist design of high‐fidelity micron‐scale two‐dimensional collagen micropatterns printed on inert and mechanically tunable^[^ ^83^, ^84^ ^]^ polyacrylamide hydrogels. Although the micropatterns are printed on flat two‐dimensional surfaces and do not fully capture the properties of the true three‐dimensional collagen matrices, the mesenchymal cell response to the micron‐scale anisotropic 2D adhesion cues is substantially faithful to cell behaviors observed in 3D fibrillar matrices.^[^ ^28^ ^]^

The used soft and rigid collagen grids represent the 5 kPa to 50 kPa mechanical range measured in vivo for human breast structures,^[^ ^31^ ^]^ and for human breast cancer lesions.^[^ ^32^ ^]^ We also note that pairwise comparison of corresponding MDA‐MB‐231 cell dimensions between soft and rigid collagen grids for each individual type of treatment displays a marginal, often statistically insignificant difference (Figure S2c, Supporting Information), demonstrating that our major blebbistatin treatment has similar effects for cells on soft and rigid grids. Indeed, it has been previously established that mesenchymal cells' mechanobiological and migratory responses to the highly structured, fibril‐like, spatially anisotropic adhesion configurations are principally different from the continuous, 2D solid, uniform cell‐surface adhesion, routinely used to study cell adhesion and migration.^[^ ^28^ ^]^ For example, MDA‐MB‐231 cells display an extremely limited response to the mechanical rigidity of the 1D anisotropic “fibrillar”‐like collagen‐1 adhesion signals.^[^ ^13^, ^15^ ^]^

The interplay between actomyosin and microtubules creates complex locomotion responses to the collagen guidance cues in metastatic MDA‐MB‐231 cells, as well as other mesenchymal‐like cancer cells.^[^ ^13^, ^79^ ^]^ While original reports suggest that actomyosin motors are principal for cell‐ECM alignment and migration within fibrillar collagen matrices,^[^ ^15^, ^53^, ^85^ ^]^ more recent reports indicate that actomyosin‐driven cytoskeletal contractility is either insufficient or impeding the cell motility.^[^ ^16^, ^17^, ^54^, ^86^ ^]^

Instead, the observed morphological conformity of low contractile cells to the substrate^[^ ^54^ ^]^ is linked both to the fluid‐like polymerization dynamics of the Arp2/3‐branched F‐actin network and to the intact microtubule dynamics.^[^ ^13^, ^15^, ^28^ ^]^ Similarly, human dermal fibroblasts switch to the low‐contractility “dendritic”‐like protrusion in 3D collagen matrices, which relies on the presence of intact and dynamic microtubules.^[^ ^87^ ^]^ Moreover, the studies in fibrosarcoma cells specifically attribute the branching of low‐contractile protrusions along 3D collagen matrix fibers to the dynein activity.^[^ ^17^ ^]^

Indeed, dyneins mechanically slide the microtubules along the advancing axon toward the neuron's growth cone complex.^[^ ^88^ ^]^ Dynein processivity within the cell cortex also mechanically repositions the entire MT apparatus and MTOC at the cell's center.^[^ ^46^, ^89^ ^]^ Thus, a tight mechanical and structural cooperation between the microtubules and the F‐actin cytoskeleton, which is mechanically powered by dynein motors,^[^ ^14^, ^17^, ^41^, ^88^ ^]^ indicates that a similar mechanism may be utilized during metastasis.

Mechanistically, our data show that the combined inhibition of myosin and dynein motors abrogates cell motility by inducing the detachment of cells (Movie S5, Supporting Information). Therefore, it is possible that simultaneous loss of myosin contractility and dynein motricity prevents force‐dependent activation of mechanosensitive focal adhesion proteins that are collectively termed as the “molecular clutch.”.^[^ ^90^ ^]^ It is also possible that inhibition of the actomyosin contractility with blebbistatin, similarly to the myosin IIA deficiency, will stabilize microtubules, shifting the balance between actomyosin and microtubules towards microtubules.^[^ ^91^ ^]^ For the mesenchymal‐like cells, the rate of motility may increase after the inhibition of myosin contractility.^[^ ^92^ ^]^ Similarly, for the epithelial‐like cells with a typical apical‐basal polarity, the blebbistatin‐induced relaxation of actomyosin may also induce migratory phenotype, which is attributed to the symmetry‐breaking event and re‐orientation of the apical‐basal cell axis towards the front‐end cell polarization.^[^ ^67^ ^]^ Moreover, our observation of cell behavior upon individual treatments are consistent with the study suggesting that dyneins and myosin motors are mechanically interconnected and may generate antagonistically counterbalanced, oppositely directed forces on microtubules.^[^ ^93^ ^]^

Specifically, if dynein‐dynactin complexes crosslink microtubules to the F‐actin, then the inhibition of non‐muscle myosin II motors, that often pull such F‐actin filament in the opposite direction, will reduce the counterbalancing forces acting at dynein and trigger a stronger MT processing. Therefore, assuming that the majority of microtubules within a cell protrusion are of uniform polarity, blebbistatin will increase the overall coherence of dynein‐generated forces. Additionally, if dynactin serves as a pseudo‐F‐actin filament, providing the nucleation seed for Arp2/3 complex assembly^[^ ^43^ ^]^ that, in turn, nucleates the branched F‐actin network around the microtubules which, in turn, acts as an adaptor between microtubules mechanosensitive focal adhesion proteins, *i.e*., molecular clutch, then the inhibition of myosin contractility could be compensated by the upregulated dynein motricity.

As for inhibition of dynein activity with dynarrestin, if dynein‐dynactin complexes resist myosin contractility within isotropic branched F‐actin network by crosslinking microtubules to microtubules and microtubules to actin filaments, then inhibition of dynein motricity could facilitate an overall enhancement of the effective myosin contractility. Thus, dynerrestin‐induced increase of the actomyosin cortex contractility, depending on the cell type and microenvironment, may to some degree be detrimental to cell spreading, formation of cell protrusions, and migration. However, if both myosin contractility and dynein motricity are inhibited, the mechanosensitive activation of the molecular clutch complex is likely to fail, which will manifest in the detachment of cells from the underlying surface.

Based on these studies, we suggest that microtubules and dyneins complement or even, in certain instances, may substitute actomyosin contractility, providing mechanical forces for cell adhesion and for protruding along the collagen guidance cues, fueling the cancer cell metastasis through crowded microenvironments. Subsequently, we show that for both metastatic mesenchymal MDA‐MB‐231 and non‐metastatic partially mesenchymal MCF‐7 cells, the migration is intertwined with the dynein‐microtubules mechanics, albeit to different extents.

We suggest that in the metastatic mesenchymal breast cancer cells, the dynein, dynactin, and microtubules form a coherent force‐generating and transmitting system that is sufficient to drive cell migration and contact guidance along the guidance cues of the collagen grids. In the nonmetastatic partially mesenchymal cells, dynein motricity facilitates cell adhesion and migration via the numerous yet incoherent radial protrusions. Moreover, the insufficient dynein motricity provides only limited engagement of MCF‐7 cells with the underlying collagen matrix, resulting in the lack of cell contact guidance and the low efficiency of migration of these cancer cells.

In addition to the orthogonal experiment with non‐metastatic breast cancer cells, we show that metastatic migration on collagen grids may also depend on mutual mechanical counterbalancing of dynein and kinesin‐1 microtubule‐associated motors, reported for the MDA‐MB‐231 cells.^[^ ^94^ ^]^ Dynein motors display (‐)‐end‐oriented MT processivity,^[^ ^95^ ^]^ while conventional kinesin‐1 displays an opposite (+)‐end‐oriented MT processivity.^[^ ^96^ ^]^ Our data are consistent with the idea that regulation of dynein versus kinesin‐1 mechanical antagonism may have a profound effect on the microtubules organization, cell contact guidance, and responsiveness to the adhesion cues through reorganization of the microtubules and their steric interactions with the surrounding microenvironments.^[^ ^13^, ^14^ ^]^

Finally, to study metastasis in tissue‐like settings, we use 3D GHS scaffolds that capture multiple biomechanical aspects of native tissue. Specifically, the granular hydrogels provide integrin‐activation by RGD peptides, tunable local microenvironment stiffness without compromising the porosity,^[^ ^97^, ^98^ ^]^ and confining interstitial void spaces among microgels. The interstitial void spaces enable facile nutrients, oxygen, and cellular products to exchange with the cell culture medium,^[^ ^99^ ^]^ resulting in a physiological‐like gradient that stimulates the migration of cancer cells inside the porous scaffold.^[^ ^99^, ^100^ ^]^ As opposed to 2D contact guidance along adhesion cues, the 3D scaffolds show that migration of metastatic and non‐metastatic breast cancer cells in confinement has more stringent requirements for the activity of dynein and myosin motors. Our results show that both actomyosin and dynein contractilities are required for confined migration, as upon administration of blebbistatin or dynarrestin, migration of cancer cells is restricted to initial cell infiltration length.

## 4 Experimental Section

### Microarray Data Analysis

Survival of breast cancer patients upon overexpression of dynactin (subunit‐5, DCTN5) and dynein (heavy chain subunit‐1, DYNC1H1) was analyzed using the microarray databases available at Human Protein Atlas (HPA). Relative expressions of DCTN2 and −5 in metastasis were analyzed using the microarray databases available at “R2‐Genomics and Visualization Platform” using inbuilt “MegaSampler” tools. Similarly, relative expressions of DCTN2 in normal versus tumors; and in different cancer stages were analyzed using the microarray databases available at R2‐Genomics and Visualization Platform using inbuilt “MegaSampler” tools. System selected cut‐offs and statistical calculations were accepted in each analysis.

### Cell Experiments

Human triple‐negative breast adenocarcinoma MDA‐MB‐231 cells (ATCC CRM‐HTB‐26) and human breast adenocarcinoma MCF‐7 cells (ATCC HTB‐26) were cultured and proliferated in Dulbecco's Modified Eagle Medium (DMEM), completed with 4.5 g L^−1^ D‐glucose, L‐glutamine,110 mg L^−1^ sodium pyruvate (Corning Cellgro, Cat#10013CV) and 10% heat‐inactivated FBS (HyClone, Cat#SH30071.03H) at 37°C in 5% CO_2_. All live cell imaging and treatments were conducted in glass‐bottom 35 mm cell culture dishes (MatTek Corp., Cat#P35G‐1.5‐14‐C) using active (‐)‐Blebbistatin enantiomer at working concentration of 25 µM (Sigma, Cat#203 391), Kinesore at 50 µM final treatment concentration (Sigma, Cat#SML2361), Dynarrestin at 50 µM cell treatment concentration (Tocris, Cat#6526), or dimethyl sulfoxide (i.e., DMSO), as an empty vehicle control (Sigma, Cat#472 301).

Cells were seeded at low or medium (1 × 10^5^–5 × 10^5^ cells mL^−1^) density onto micropatterned collagen grids, mounted on the glass‐bottom dishes, in the complete DMEM. For confocal immunofluorescence imaging, cells were fixed with a fixative solution, formulated as follows: 3% Paraformaldehyde (BeanTown Chemical, Cat#50 071 991), 0.25% Triton X‐100 (Roche, Cat#11 332 481 001), 0.2% Glutaraldehyde (Sigma‐Aldrich, Cat#340 855) in 1×PBS without Ca^2+^ and Mg^2+^ (Corning, Cat#21‐040‐CM) at 37 °C for 15 min. Then samples were rinsed twice in 1×PBS without Ca^2+^ and Mg^2+^ for 10 min each cycle, then quenched in cold (i.e., *t* = 4°C) cytoskeletal buffer (i.e., 10 mM MES (Sigma‐Aldrich, Cat#M3671‐50G), 150 mM NaCl (Flinn Scientific, Cat#S0062), 5 mM EGTA (Sigma‐Aldrich, Cat#4100‐50GM), 5 mM MgCl_2_ (Fisher Scientific, Cat#M33‐500), 5 mM Glucose (Acros, Cat#41095‐0010), pH 6.1) with freshly added sodium borohydride (1 mg mL^−1^; TCI, Cat#S0480) for 15 min on ice. The quenched samples were then rinsed three times in 1×PBS, 5 min each cycle, then blocked with 1% BSA (Fisher Bioreagents, Cat#BP9704‐100) and immunostained.^[^ ^101^ ^]^ For immuno‐fluorescent microtubules staining, we utilized primary antibodies against β‐tubulin (Sigma, Cat#T7816) diluted in 1% BSA PBS, incubated with the samples for 1 h at ambient temperature of 20°C. Consequently, primary antibodies‐labeled structures were stained with Alexa‐Fluor‐conjugated secondary antibodies (Thermo Fisher) at the final concentration of 5 µg mL^−1^, 1 h in 1% BSA PBS at ambient temperature. F‐actin was selectively labeled with fluorescent phalloidin (Phalloidin‐ATTO 647N conjugate, Millipore‐Sigma, Cat#65 906; 10 U mL^−1^). Cell chromatin was labeled with 1:1000 Hoechst solution (Tocris, Cat#5117). Samples were mounted using 90% Glycerol (Sigma, Cat#G5516) in 1×PBS.

For instant structured illumination microscopy (iSIM), we fixed cells with −20°C methanol in the freezer for the duration of 5 min. Methanol‐fixed cells were rinsed with 1×PBS and additionally fixed with 4% Paraformaldehyde (Sigma, Cat#P6148) for the duration of 15 min at room temperature. PFA‐fixed cells were then rinsed with 1% BSA in PBS, followed by 60‐min‐long blocking with 1% BSA (Fisher, Cat#BP9704) in PBS (Thermo Fisher, Cat#10 010 023). For immunofluorescence staining, we utilized following primary antibodies: rabbit anti‐α‐tubulin (Abcam, Cat#ab18251), mouse anti‐dynactin 1 (1:250, BioLegend, Cat#867 602), mouse anti‐dynein (1:1000, Millipore, Cat#MAB1618), mouse paxillin (Novus Biologicals, Cat#P49023), diluted in 1% BSA PBS. The duration of the incubation with any of the listed primary antibody solutions was 1 h at room temperature. Similarly, labelings with Alexa‐Fluor‐conjugated secondary antibodies (Thermo Fisher) were performed at their final concentration of 5 µg mL^−1^ for the duration of 1 h in 1% BSA PBS at room temperature. After washing out the excess secondary antibodies, chromatin was labeled with 1:1000 Hoechst solution (Tocris, Cat#5117). We mounted samples using 90% Glycerol (Sigma, Cat#G5516) in 1×PBS. Instant structured illumination microscopy (iSIM) was performed using the custom‐built instant structured illumination microscope system (VisiTech Intl, Sunderland, UK) equipped with an Olympus UPLAPO‐HR ×100/1.5 NA objective, two Flash‐4 scientific CMOS cameras (Hamamatsu, Corp., Tokyo, Japan), an iSIM scan head (VisiTech Intl, Sunderland, UK), and a Nano‐Drive piezo Z stage (Mad City Labs, Madison, WI). The iSIM scan head included the VT‐Ingwaz optical de‐striping unit. Image acquisition and system control was done using MetaMorph Premiere software (Molecular Devices, LLC, San Jose, CA). Images were deconvolved with an iSIM‐specific commercial plugin from Microvolution (Cupertino, CA) in FIJI.

For flow cytometry analysis of intracellular dynactin‐1, MCF‐7, and MDA‐MB231 cell samples were resuspended in TrypLE™ Express Enzyme (Gibco, Cat#12 604 013), pelleted by centrifugation 200xg for 5 min, resuspended and fixed in 4% Paraformaldehyde in PBS for 15 min at room temperature. PFA‐fixed samples were rinsed twice with 1% BSA PBS using centrifugation at 500x g for 5 min and incubated for 60 min in a blocking‐permeabilization solution of 0.1% Triton X‐100 (X100, Sigma) with 1% BSA (Fisher, Cat#BP9704) in PBS (Thermo Fisher, Cat#10 010 023). Blocked cells were rinsed twice with 1% BSA PBS using centrifugation at 500x g for 5 min. Rinsed cells were separately labeled for flow cytometry identification with either Alexa Fluor Plus 405 Phalloidin (Invitrogen, Cat#A30104, 1:100) or Alexa Fluor 488 Phalloidin (Invitrogen, Cat#A12379, 1:100) in 1×PBS for 1 h at room temperature. Phalloidin‐stained cells were washed twice with 1×PBS using centrifugation, as before, resuspended in 1×PBS, and mixed in one tube. The total protein in mixed cells was stained using Alexa Fluor 568 NHS Ester (Invitrogen, Cat#A20003) at a final concentration of 0.1 mg mL^−1^ for 1 h at room temperature. The total protein‐stained cells were washed with 1×PBS three times using centrifugation, as before, and resuspended in 1% BSA in PBS with primary mouse anti‐dynactin 1 antibody (BioLegend, Cat#867 602, 1:250) for 1 h at room temperature. Cells were washed, as before, and labeled with Goat anti‐Mouse IgG (H+L) Cross‐Adsorbed Secondary Antibody, Alexa Fluor 647 (Invitrogen, Cat#A‐21235) at final concentration of 5 µg mL^−1^ in 1% BSA PBS for the duration of 1 h at room temperature. Cells were washed, as before, and immediately analyzed by flow cytometry.

For analysis of cell viability using CellEvent Caspase‐3/7 detection reagent, the MDA‐MB‐231 cells were resuspended in fresh media with 25 µM of Blebbistatin and 5 µM of Caspase‐3/7 detection reagent (Invitrogen, Cat# C10423). Cells were seeded on a rhomboid collagen grid (*G*′ = 55 kPa) and at 24 h re‐supplied with fresh prewarmed media containing 25 µM of Blebbistatin and 5 µM of Caspase‐3/7 detection reagent. Data for analysis of cell viability were collected by live cell imaging for the duration of 48 h. Live cell imaging was performed on epifluorescent Leica DMi8 AFC microscope (Leica, Germany), equipped with a temperature (37°C), CO_2_ (5%), and humidity‐controlled chamber (OkoLab, Italy) at 20× magnification. Image acquisition was performed using Leica LAS X software (Leica, Germany). For image analysis, we used ImageJ/FIJI. Figures were composed as before, i.e., using unmodified LAS X ‐generated TIFF images with Adobe Illustrator CC 2021 (Adobe).

For over‐expression of the CC1 domain of p150 (Glued) in MDA‐MB‐231 cells, the CC1 domain of human p150 (Glued) was assembled commercially (Vector Builder, Cat# pLV[Exp]‐CMV>EGFP/{Human P150Glued CC1} (VB230524‐1483jpz)). For viral production, the CC1 DNA was isolated and purified from a manufacturer‐prepared glycerol stock using ZymoPURE II Plasmid Midiprep (ZymoResearch, Cat#D4201), following the kit's manufacturer protocol. For viral packaging, two separate tubes were prepared: tube 1 was prepared with 500 µL of room‐temperature Opti‐MEM (Gibco, Cat#51985‐034), 9.2 µg of packaging plasmid (pCMVR8.74), 2.8 µg of envelope plasmid (pMD2.G), and 11 µg of the CC1 viral plasmid; tube 2 was prepared with 500 µL of Opti‐MEM and 30 µL of Lipofectamine2000 (ThermoFisher, Cat#11 668 019). Both tubes were left to incubate separately at room temperature for 5 min before being mixed together. After the two tubes were combined, the mixture was left to incubate at room temperature for 30 min. Then the plasmid mixture was added to 9 × 10^6^ HEK293T cells in a 10‐cm dish and cultured in DMEM (Gibco, Cat#10569‐010) with 20% FBS (Gibco, cat no. 10438‐026). The cells were then incubated at 37°C for 48–72 h. After 48–72 h, the viral supernatant was harvested and used to infect the MDA‐MB 231 cells. For viral infection, 3 × 10^5 of MDA‐MB‐231 cells were seeded into individual wells of a 48‐well plate. The appropriate volume of DMEM with 10% FBS was added to the wells and then the CC1 viral particles were added at 1/10 of the final volume. After 5 days of incubation, the cells were then sorted for positive GFP expression. The GFP‐positive cells and control cells were seeded in 1:1 ratio on rhomboid collagen grids (*G*′ = 55 kPa), either with or without 25 µM of blebbistatin. Subsequently, cells that were allowed to attach without blebbistatin were treated with 25 µM of blebbistatin after overnight incubation (*t* = 16 h). Live cell imaging of cell attachment, spreading, and crawling was performed on the epifluorescent Leica DMi8 AFC microscope as described before.

### High Precision Micropatterning

A step‐by‐step methodological instruction and a detailed protocol for high‐fidelity, sub‐micron precision polyacrylamide gels micropatterning with various ligands, to include collagen type‐1, were described elsewhere.^[^ ^84^ ^]^ Briefly, in order to prevent van‐der‐Waals and common deformation effects that routinely cause the collapse of soft lithographic stamp (i.e., soft regular PDMS (rPDMS)) onto the printed glass surfaces, we replaced rPDMS with a composite –, i.e., soft cushioning rPDMS layer, veneered with a ≈0.5 mm hard noncollapsing PDMS (hPDMS, see “hPDMS formulation” section).^[^ ^13^, ^102^ ^]^ We cast micro‐printing surfaces with the commercially premade silicon crystal molding matrix (Minnesota Nano Center, University of Minnesota) by coating the passivated molds with ≈0.5 mm layer of hPDMS, followed by 30 min‐long curing at 70°C. Next, a 5–6 mm‐thick layer of degassed rPDMS liquid premix is poured atop of the solidified hPDMS layer (rPDMS; 1:5 curing agent/base ratio, Sylgard‐184, Dow Corning), then baked at 70°C for ≈1 h. The resulting cured composites were then released from the molding surfaces, and carefully cut into square blocks of 1 × 1 cm in size.

Collagen type‐1 protein is not directly printable via the means of soft micro‐contact lithography due to its gelation. Thus, to prepare the collagen type‐1 micro‐patterns, we microprinted anti‐collagen type‐1 polyclonal rabbit Ab (AbCam, Cambridge, UK, Cat#ab34710; RRID:AB_731 684), pre‐conjugated with a biotin tag, ((+)‐biotin *N*‐hydroxysuccinimide ester, Sigma–Aldrich, Cat#H1759; commercial protocol) and a fluorescent tag (Alexa Fluor succinimidyl esters, Invitrogen, Molecular Probes, Cat#A20000, Cat#A20003; as per supplied protocol) on a clean (i.e., dust‐free) intermediate coverglass (FisherFinest Premium Cover Glass; #1, Cat#12‐548‐5 P). Soft lithographic micro‐printing was executed by incubating the composite micro‐stamp's surfaces with the 0.2 mg mL^−1^ biotin and fluorophore pre‐conjugated α‐collagen‐1 antibody, reconstituted in 1×PBS (40 min, 37°C) in a humid chamber. The coated stamps were rinsed in deionized water, then blow‐dried with air. The dried stamps were then gently applied to the intermediate glass surfaces with their antibody‐coated printing surfaces, then 100 g weight was placed atop the stamp to ensure proper contact between the stamping surface and the intermediate glass for 1 min and then removed. The quality of the resulting micropatterns was examined with the epifluorescent microscope (ThermoFisher, EVOS M5000, Cat#AMF5000).

A 35 mm cell culture plastic dishes with 20 mm circular cutouts, sealed with the glass bottom (MatTek Corp., Ashland, MA, Cat#P35G‐1.0‐20‐C) were chemically activated to covalently cross‐link with polyacrylamide (PAA) gels, i.e., coated with 3‐(trimethoxysilyl)propyl methacrylate (Sigma‐Aldrich, Cat#6514, as per commercial protocol). Small volumes (e.g., ≈5 µL) of PAA premix (see the “PAA elastic gel premix” section) of the projected rigidity G′ = 8.6 or 55 kPa,^[^ ^83^ ^]^ complemented with 5% streptavidin‐acrylamide (ThermoFisher, Cat#S21379), were “sandwiched” between the activated dish's glass bottom and the micropatterned intermediate glass upon adding a curing catalyst (aminopropyltriethoxysilane (APS)). After PAA curing is complete (≈5 min), the resulting PAA “sandwiches” were incubated in hypotonic (i.e., deionized) water for 1 h at room temperature (20°C) to ensure PAA gel osmotic swelling and its gentle deformation for easier intermediate glass release from the PAA layer. The resulting PAA gels, attached to the glass bottoms and with the cross‐linked antibody micropatterns, were quality‐controlled and selected by examining the micropatterns’ quality with an epifluorescent microscope. The selected micropatterns with α‐collagen‐1 Ab were then incubated with 1 mg mL^−1^ rat monomeric collagen‐1 (Corning, NY, Cat#354 249) in cold PBS (4°C, 12 h), then rinsed three times with cold PBS, and utilized for experiments.

hPDMS formulationhPDMS premix composition is as follows: 3.4 g of VDT‐731 (Gelest, Inc., Cat#VDT‐731), 18 µL of Pt catalyst (Platinum(0)−2,4,6,8‐tetramethyl‐2,4,6,8‐tetravinylcyclotetrasiloxane complex solution) (Sigma–Aldrich, Cat#479 543), 5 µL of cross‐linking modulator 2,4,6,8‐Tetramethyl‐2,4,6,8‐ tetravinylcyclotetrasiloxane (Sigma–Aldrich, Cat#396 281). The resulting mixture was mixed in a 50 mL conical bottom centrifuge tube on the vortex mixer for at least 30 s, then 1 g of HMS‐301 (Gelest, Inc., Cat#HMS‐301) was added immediately before use and mixed for 30 s on a vortex mixer.^[^ ^102^, ^103^ ^]^

### PAA Elastic Gel Premix

To prepare the customized PAA premixes with projected gel rigidity, we utilized 40% acrylamide (40% AA) as a base (BioRad, Cat#161–0140), and 2% bis‐AA (BioRad, Cat#161–0142) as a cross‐linker.^[^ ^83^, ^104^ ^]^ The PAA premixed was also supplemented with streptavidin‐acrylamide (Thermo Fisher, Cat#S21379) to the final concentration of 0.133 mg mL^−1^ to facilitate PAA gels cross‐linking with biotin‐conjugated anti‐collagen antibodies (i.e., micropatterns). For the preparation of 5 µL of *G*′ = 8.5 or 55 kPa PAA gel premixes respectively, the components were mixed as follows: 40% AA – 2.34 or 2.25 µL; 2% bis‐AA – 1.88 or 2.25 µL; 2 mg mL^−1^ streptavidin‐acrylamide – 0.33 µL for both rigidities; 10×PBS – 0.5 µL for both rigidities; deionized milli‐Q water – 1.117 µL for both rigidities; TEMED – 0.01 µL for both rigidities. The premix solutions were ultrasonicated to remove gas, and then stored at 4°C before use. To initiate the polymerization of PAA premixes 0.1 µL of 10% APS was added to 5 µL of PAA premix immediately prior to the PAA gel casting procedure.

### Nano‐Textured Surfaces Assay

Clean textured polystyrene nano‐surface cell culture dishes (NanoSurface Biomedical, Seattle, WA, Cat# ANFS‐0001) were treated in an ozone plasma chamber for 2 min to activate their surface. Immediately after the treatment the dished were incubated with 1 mg mL^−1^ rat monomeric collagen‐1 (Corning, NY, Cat#354 249) in cold PBS (4°C, 12 h), rinsed three times with sterile PBS and used for the cell seeding, adhesion and spreading assays, followed by the treatments, fixation, and imaging.

### Western Blot

For cell extract preparation, confluent MCF‐7 and MDA‐MB 231 cells were washed twice with PBS. A mixture of RIPA Buffer‐2 (2X, ThermoScientific,Cat#J60629EQE) and cOmplete, Mini, EDTA‐free Protease Inhibitor Cocktail (Roche Diagnostics, Cat#11 836 170 001) was added to uniformly cover the dish surface. The dishes were then incubated on ice for 10 min. After, the cell extract was scraped off the dish and collected in a microcentrifuge tube, sonicated for 30 s at 50% pulse, and centrifuged at 21 000 x g for 15 min. Following centrifugation, the supernatant was collected and stored at −20°C. The Pierce BCA Protein Assay Kit (Cat#23 227) was used to determine the protein concentrations of the cell extracts. For use in western blot, a solution of 1X NuPAGE™ LDS Sample Buffer (4X, Invitrogen, cat no. NP0008), 1X NuPAGE Sample Reducing Agent (10X, Invitrogen, Cat#NP0009), and 1 µg µL^−1^ cell extract was prepared. This solution was warmed at 100°C for 10 min and then stored at −20°C.

After removing the cell extract solution from the freezer, the samples were heated at 70°C for ten minutes. The 10 µg of samples and the PageRuler Plus Prestained Protein Ladder (Protein Biology, Cat#26 619) were then loaded onto a NuPAGE 4–12% Bis‐Tris Gel with 12 wells (Invitrogen). The gel was run with 1X NuPAGE MOPS SDS Running Buffer (20X, Invitrogen, Cat#NP0001‐02) and ran at 150 V for 75 min. The gel was then transferred to a low‐fluorescence PVDF transfer membrane (ThermoFisher), which was placed into iBlot PVDF Mini Stack, replacing the PVDF membrane that was pre‐packaged into stack. Next, the Mini Stack was moved into the iBlot2 instrument, where the protein transfer occurred at 20 V for seven minutes. Following the transfer, the membrane was blocked in Intercept TBS Blocking Buffer (LI‐COR) for 4 h, shaking at room temperature.

After blocking, the membrane was cut into three sections, one for each protein (Cell Signaling). Each membrane piece was then incubated overnight at 4°C, shaking, with the target protein's respective antibody. The following primary antibodies were used: mouse anti‐dynactin 1 (1:250, BioLegend, Cat#867 602), mouse anti‐dynein (1:1000, Millipore, Cat#MAB1618), and mouse anti‐beta actin (1:1000, Abcam, Cat#ab8226). The antibodies were diluted with Intercept Antibody Diluent T20 TBS (LI‐COR, Cat.#927‐65001). After overnight incubation, the membranes were washed with 0.05% TBS‐T three times, 5 min each, shaking at room temperature.

Next, the membrane pieces were incubated for one hour at room temperature, shaking, with the respective secondary antibody. For the mouse‐isotype primary antibodies, IRDye 680RD Goat anti‐Mouse Secondary Antibody (1:20 000, LI‐COR, Cat#926‐68070) was used. Antibody was diluted with the Intercept Antibody Diluent T20 TBS. Following the incubation, the membranes were again washed with 0.05% TBS‐T three times, five minutes each, shaking at room temperature. The membranes were then imaged on a LI‐COR Odyssey CLx and analyzed via ImageStudio (version 5.2).

### Cell Migration Assay

The confocal imaging was performed on the Leica TCS SP8 laser scanning confocal microscope with LIAchroic Lightning system and LAS X Lightning Expert super‐resolution capacity, 405, 488, 552, and 638 nm excitation diode lasers, with 40×/1.3 oil immersion objective (Leica, Germany). The scanning settings were optimized with Nyquist LAS X function with HyD2.0‐SMD excitation sensors, at the regular pinhole size of 0.85 AU, and the scanning frequency at 100 Hz. Each frequency channel was scanned in sequence in order to avoid signal bleeding between the channels. Morphometric analysis was performed automatically and/or manually utilizing LAS X (Leica, Germany) and ImageJ/FIJI. Figures were composed using Adobe Illustrator CC 2023 (Adobe).

### GelMA Synthesis

GelMA synthesis was conducted based on our previously established protocol.^[^ ^97^, ^98^ ^]^ Briefly, 2.5 mL of methacrylic anhydride (Sigma, MO, USA) was added to DPBS (Gibco, MA, USA) containing 10% w/v of porcine skin gelatin powder (≈300 g Bloom, Type A, Sigma, MO, USA) dropwise and allowed to react for 2 h at 50°C while stirring. Then, DPBS was added to the solution (2:1 volume ratio) to stop the reaction. The solution was dialyzed for 10 days against ultrapure Milli‐Q water (Millipore Corporation, MA, USA) at 40°C using standard grade regenerated cellulose dialysis membranes (Spectra/Por 4 with Mw cutoff = 12–14 kDa, Spectrum Laboratories, NJ, USA). The final solution was then filtered using vacuum filtration (pore size = 0.2 µm, VWR, PA, USA). The GelMA solution was then transferred to 50 mL centrifuge tubes (Celltreat, MA, USA) and subsequently frozen at −80°C while being placed in a sideways position. After 48 h, the frozen GelMA was lyophilized for 3 days to yield solid GelMA.

### Bulk Hydrogel Scaffold Fabrication

Bulk hydrogel scaffolds were fabricated using different GelMA concentrations and varying light exposure times. Soft or stiff scaffolds were prepared by dissolving GelMA in a photoinitiator solution (0.1% w/v of lithium phenyl‐2,4,6‐trimethylbenzoylphosphinate (LAP, Sigma, MO, USA) in DPBS) at 37°C at the concentrations of 50 mg mL^−1^ or 150 mg mL^−1^, respectively. The dissolved GelMA was then poured into a cylindrical mold (diameter = 8 mm and height = 1 mm). The molded GelMA solution was then placed in a humidity chamber made from a petri dish with wet Kimwipes and stored at 4°C overnight to form a physically crosslinked gel while protected from light. Finally, physically crosslinked molded GelMA hydrogels were exposed to light (wavelength = 395–405 nm and intensity = 15 mW cm^−2^) for 1 min (5% w/v GelMA solution) or 2 min (15% w/v GelMA solution) to yield the soft or stiff scaffolds.

### GelMA Microgel Fabrication

A step emulsification microfluidic device was fabricated for high throughput microgel production as described previously.^[^ ^97^, ^98^ ^]^ For soft or stiff GelMA microgels, 50 mg mL^−1^ or 150 mg mL^−1^ of GelMA solution was prepared in 0.1% w/v photoinitiator solutions, respectively. Novec 7500 engineered fluid (3 M, MN, USA) supplemented with Pico‐surf (2% v/v) (Sphere Fluidics, Cambridge, UK) was prepared for the continuous phase. The GelMA and Pico‐surf™ solutions were then injected into the microfluidic device using syringe pumps (PHD 2000, Harvard Apparatus, MA, USA). The temperature was maintained at 35–40°C using a space heater. Once collected in microcentrifuge tubes, microgel suspensions were stored at 4°C to physically crosslink.

### GHS Fabrication

While protected from light, oil and surfactant were removed from the microgel suspension using 1H,1H,2H,2H‐perfluoro‐1‐octanol (20% v/v, Alfa Aesar, MA, USA) in Novec 7500 engineered fluid. To this end, the solution was added to the suspension (1:1 volume ratio) and vortexed for 10–20 s, followed by centrifugation at 325×g for 15 s. Then, 400 µL of photoinitiator solution (LAP in DPBS, 0.1% w/v) was added to the suspension, vortexed for 10 s, and centrifuged at 325×g. This step was followed by the addition of 400 µL of photoinitiator solution, vortexing for 10 s, and centrifugation at 1310×g. The supernatant was decanted, and microgel suspension was pipetted into a mold (diameter = 10 mm and height = 3 mm), using a positive displacement pipette (Microman E M100E, Gilson, OH, USA). The molded microgels were then placed in a chamber and exposed to light (395–405 nm and 15 mW cm^−2^) for 1 min or 2 min to form GHS made up of soft or stiff microgels (termed as soft GHS or stiff GHS), respectively.

### Rheological Characterizations

The rheological properties (storage and loss moduli) of bulk hydrogel scaffolds, representing the individual microgels, were characterized by placing the scaffolds (diameter = 8 mm and height = 1 mm) between two sandblasted parallel plates (upper plate diameter = 8 mm, bottom plate diameter = 25 mm) in an AR‐G2 rheometer (TA instrument, DE, USA). Once sandwiched between the plates, the amplitude sweep test was conducted on samples at a constant frequency of 1 rad s^−1^ to find the linear viscoelastic region (LVR). Then, a frequency sweep test was performed on samples at a constant oscillatory strain of 0.1% (within the LVR) from 0.1 to 100 rad s^−1^.

### Pore Characterization of GHS

GHS was incubated in a fluorescein isothiocyanate–dextran solution (Sigma, MO, USA, with an average molecular weight of 2 MDa) in Ultra‐Pure Milli‐Q water (15 µM). The scaffolds were then imaged using a Leica DMi8 Thunder microscope (Germany). The Leica application suite X (LAS X, version 3.7.4.23463) software was used to construct 3D images from high‐magnification (250×) *z*‐stacked images (141 z‐slices, increment size = 0.502 µm). The void fraction was then measured as the fraction of occupied void space over the total volume, using LAS X software. Also, for pore detection and reporting equivalent median pore diameter, a MATLAB code was developed and used, wherein 2D fluorescence microscopy images were thresholded, and the pores were detected and masked. The areas of detected pores were obtained and converted to equivalent‐area circles, from which the diameters were calculated. The median of the equivalent diameter distribution was reported.

### Cell Migration Assay

Cell migration was analyzed using the MDA‐MB‐231 or MCF‐7 cell lines labeled with CellTracker Green 5‐chloromethylfluorescein diacetate (CMFDA) Dye (Invitrogen, MA, USA). Briefly, 50 µg of the dye was reconstituted in 20 µL of DMSO (Sigma. MO, USA). Then, the dye solution was diluted in DMEM (Gibco, MA, USA) (1:1000 volume ratio), and 10 mL of dye‐containing culture media was added to the cell culture flasks (VWR, PA, USA). Cells were incubated under a 5% v/v CO_2_ atmosphere at 37°C for 30 min. Then, culture medium was replaced with DMEM, supplemented with 10% v/v FBS (Cytiva, MA, USA) and 1% v/v antibiotic (10 000 U mL^−1^ penicillin G, 10 000 µg mL^−1^ streptomycin, 25 µg mL^−1^ amphotericin B, Cytiva, MA, USA). After 3 h of incubation in antibiotic‐containing DPBS (1% v/v) at room temperature, scaffolds were transferred to a non‐treated 24‐well cell culture plate (VWR, PA, USA). Then, 20 µL of cell suspension (5 × 10^6^ cells per mL in complete cell culture media) was added on top of the scaffolds. After topical seeding, scaffolds were incubated at room temperature for ≈30 min, and then complete cell culture media with or without cell contractility inhibitors (final working concentration of 50 µM) were added to each well, and the culture dish was placed in an incubator (5% v/v CO_2_ atmosphere at 37 °C). After 72 h, samples were sectioned through their sagittal plane, using a razor blade (VWR, PA, USA) and imaged using the Leica DMi8 Thunder microscope at an excitation wavelength and emission wavelength of 470 nm (blue) and 550 nm (green), respectively. Then, cell migration length was measured throughout the scaffold at different points using the ImageJ software (FIJI, version 1.53t, NIH, MD, USA),^[^ ^105^ ^]^ and the average migration length was reported.

### Quantification of Cell Migration on Anisotropically Aligned Collagen Patterns

The quantification analyses for Figures 3, 4, and 6; Figures S1 and S7, Supporting Information were performed with an automated pipeline based on in‐house MATLAB scripts. The pipeline includes the following steps:

To identify individual cells in time‐lapsed‐based phase contrast images, we: 1) evened the background by subtracting images smoothed by Gaussian kernel with a specified standard deviation (20 pixels for our 2048×2048 data) from the original images; 2) identified local variations (cells, cell edges, and protrusions) with the standard deviation (STD) filter using 5‐by‐5 neighborhood; 3) segmented the resulting STD map; 4) corrected for over‐segmentation by the erosion filter using a circular structural element of radius 15 pixels; 5) filtered out very small and very large objects (<10 and >7000 pixels, respectively); 6) recorded the coordinates of the centroids of the remaining objects (cells). To choose the proper threshold values for step (3), we run an optimization routine that finds a threshold value maximizing the number of identified cells in each image at the end of the processing.

Next, we used a pipeline for extracting cell tracks. This pipeline consists of 1) matching cells between consecutive time frames; 2) correcting for flickering events; and 3) correcting for track cloning. In the first step, we match each cell at time *t* with the nearest cell at time *t*+1 within a circle of radius *R*_max_ (20 pixels in for our data). Because cells in our data may have very complex geometries with cell bodies being on the edge of the algorithm's detection margins, it is possible that tracks become interrupted by cell “disappearance” for one‐time point (a.k.a. flickering). In step (2), we use an algorithm that detects such events based on the proximity of tracks ending at time *t* and tracks starting at time *t*+2 and connects the interrupted tracks together. For the same shape complexity reason, it is possible that occasionally one cell can be detected as two closely located ones. In such cases, we can obtain two trajectories representing one cell and closely following each other. In step (3), we use an algorithm that detects such cloned tracks and eliminates them from the record.

In our data, the detected tracks accurately match the observed complex movement of the cells. However, frame‐to‐frame variations in cell shape and slight jiggling of the microscope stage contribute to a pixel‐size noise along the cell tracks. To minimize such instantaneous distortions, we smoothed all tracks by applying the running average to *x* and *y* coordinates of the track points (15 min window in 1 min steps) before calculating angular directions and velocity values along the tracks.

Based on the collected cell migration data the mean squared displacement (MSD) and directionality ratio parameters were calculated. While MSD at each point of time is calculated as the squared value of cell displacement from its position of origin at the beginning of cell tracking, the directionality ratio is calculated as the length of a straight line between the beginning and the end of the cell track divided by the distance along the track giving a value between 0 and 1. In extreme cases, cells moving in one direction along a straight line would have a directionality ratio of one, while cells moving randomly near the starting point would have a directionality ratio close to zero.^[^ ^106^ ^]^

### Statistical Analysis

For pairwise comparisons of cell migration speeds (Figures 3, 4, and 6c) and directionality ratios (Figure S1f, Supporting Information) of a control group with different perturbation conditions, we calculate p‐values using two‐sample *t*‐test as implemented by MATLAB's *ttest2* function. Computation of the other statistical data was performed with KaleidaGraph 4.5.4 (Synergy Software) and Prism 7b (GraphPad Software, Inc). The numeric p‐values are denoted on the corresponding plots. If the *p*‐value is below 0.0001, i.e., cutoff lower limit for Kaleidagraph and Prism 7b, a ‘*p* < 0.0001′ is denoted. Sample size n (i.e., number of analyzed cells) for each measured parameter is denoted on the plots. Each measured parameter is a result of three replicates (independent experiments), unless specified otherwise. Box and whisker plots reflect the first quartile, median, third quartile, and 95% percent confidence interval. Data shown as violin diagrams are distributions. Mean and median values are outlined separately.

## Conflict of Interest

There are no conflicts of interest to declare.

## Author Contributions

Y.T. and S.K. contributed equally to this work. E.D.T. and Y.T. theoretically conceptualized and conceived the project, E.D.T., Y.T., A.S.Z., A.S., and D.T. theoretically and experimentally developed the project. E.D.T., Y.T., S.K., Z.A., D.T., and A.S. designed the experimental framework of this study and conducted experiments, R.K.S. conducted the database analysis, Y.T. formulated and conducted cell biology experiments, O.P., A.N., and X.M. conducted biochemical experiments, A.S.Z. conducted imaging experiments, D.T. developed a high throughput computational algorithm for dynamically complex cell behaviors analysis, and conducted detailed data analysis, E.D.T., S.K., A.S., Y.T., and A.S.Z conducted experimental data interpretation and analysis. E.D.T. and A.S.Z wrote the manuscript, all co‐authors participated in editing the manuscript. E.D.T. oversaw all aspects of this study.

## Supporting information

Supporting Information

Supplemental Movie 1

Supplemental Movie 2

Supplemental Movie 3

Supplemental Movie 4

Supplemental Movie 5

Supplemental Movie 6

Supplemental Movie 7

Supplemental Movie 8

Supplemental Movie 9

Supplemental Movie 10

Supplemental Movie 11

Supplemental Movie 12

Supplemental Movie 13

Supplemental Movie 14

## Data Availability

The data that support the findings of this study are available from the corresponding author upon reasonable request.
