# Supplementary material for: Dynein‐Powered Cell Locomotion Guides Metastasis of Breast Cancer
Source: Adv Sci (Weinh). 2023 Sep 19;10(31):2302229. doi: 10.1002/advs.202302229 (PMC10625109; doi:10.1002/advs.202302229)
Supplement: Supplementary file 1 — Supporting Information [file ADVS-10-2302229-s009.pdf]

ADVANCED SCIENCE
Open Access

Supporting Information

for *Adv. Sci.*, DOI 10.1002/advs.202302229

Dynein-Powered Cell Locomotion Guides Metastasis of Breast Cancer

Yerbol Tagay, Sina Kheirabadi, Zaman Ataie, Rakesh K. Singh, Olivia Prince, Ashley Nguyen, Alexander S. Zhovmer, Xuefei Ma, Amir Sheikhi, Denis Tsygankov* and Erdem D. Tabdanov**

Movie legends:

Movie 1. Timelapse of MDA-MB-231 cells' migration along the combined collagen type-I micropattern: parallel 1D lanes (*left*) and 2D rhomboid grids (*right*). The mechanical rigidity of the combined micropattern is G′=8.6 kPa. The scale bar and timestamp are denoted on the video.

Movie 2. MDA-MB-231 cell migration and morphology in control conditions (+DMSO, G′=55 kPa). Collagen type-1 rhomboid grid is partially depicted in a single quadrant of the field of view. The scale bar and timestamp are denoted on the video.

Movie 3. MDA-MB-231 cell migration and morphology during low actomyosin contractility (+Blebb, G′=55 kPa). The scale bar and timestamp are denoted on the video.

Movie 4. Timelapse of MDA-MB-231 cell transition from the polygonal mode of cell-collagen grid adhesion towards the 'dendritic'-like mode of protrusion upon inhibition of actomyosin contractility with blebbistatin (*arrows*). The mechanical rigidity of the rhomboid collagen grid micropattern is G′=8.6 kPa. The scale bar and timestamp are denoted on the video.

Movie 5. Failure of the blebbistatin-induced 'dendritic'-like protrusions and MDA-MB-231 cells detachment upon inhibition of the dynein with dynarrestin (+Blebb→+Blebb+DA, G′=55 kPa). The scale bar and timestamp are denoted on the video.

Movie 6. Timelapse of microtubules' disassembly-induced disruption of dendritic protrusions and loss of structural integrity (*arrows*) in blebbistatin-pretreated MDA-MB-231 cells upon addition of 10 μM nocodazole. The mechanical rigidity of the rhomboid collagen grid micropattern is G′=8.6 kPa. The scale bar and timestamp are denoted on the video.

Movie 7. Timelapse of adhesion and protrusion dynamics in the mixed MDA-MB-231 cell population: **(1)** wild type, and **(2)** transfected with CC1-GFP. Prior to the live imaging, the cell mixture was preincubated with the collagen grids for 48 hours to allow for cell adhesion and protrusion. The mechanical rigidity of the rhomboid collagen grid micropattern is G′=55 kPa. The scale bar and timestamp are denoted on the video.

Movie 8. Timelapse of adhesion and protrusion dynamics in the mixed MDA-MB-231 cell population: **(1)** wild type, and **(2)** transfected with CC1-GFP. Cell mixture is freshly added to the collagen rhomboid grids to capture the *de novo* cell adhesion and protrusion dynamics. The mechanical rigidity of the rhomboid collagen grid micropattern is G′=55 kPa. The scale bar and timestamp are denoted on the video.

Movie 9. Compiled video-sequences for migration of MDA-MB-231 cells along soft (G′=8.6 kPa, *left*) and rigid (G′=55 kPa, *right*) collagen type-1 rhomboid grids during primary treatment sequence (+DMSO→+Blebb). The control condition (+DMSO*, top*) and low actomyosin contractility (+Blebb*, bottom*) cell migration state are shown. Cell migration tracks are highlighted as computed cell centroid displacement (*yellow tracks*). See cell detachment in (+Blebb+DA on G′=55 kPa) in Movie 5. Frame frequency (*i.e.*, frames per second, fps) is 32 fps, and each frame corresponds to 1 minute of real-time microscopy. The scale bar is 20 μm on the panels with denoted tracks. The scale bar on the zoom-in panels with stationary cell centroids is 50 μm.

Movie 10. Video-sequences for MDA-MB-231 cells migrating along the soft (G′=8.6 kPa, *left*) and rigid (G′=55 kPa, *right*) collagen grids, reversed treatment sequence (+DMSO→+DA). Control cells (+DMSO*, top*) and inhibited dynein (+DA*, bottom*) cell migration states are shown. Cell migration tracks are highlighted as computed cell centroid displacement (*yellow tracks*). Frame frequency (*i.e.*, frames per second, fps) is 32 fps, and each frame corresponds to 1 minute of real-time microscopy. The scale bar is 20 μm on the panels with denoted tracks. The scale bar on the zoom-in panels with stationary cell centroids is 50 μm.

Movie 11. Compiled migration modes sequences for migrating MDA-MB-231 cells along the soft (G′=8.6 kPa, *left*) and rigid (G′=55 kPa, *right*) collagen grids during +Blebb→Blebb+KS treatment sequence. Low actomyosin

contractility conditions alone (+Blebb*, top*) and its combination with enhanced kinesin-1 activity (+Blebb+KS*, bottom*) cell migration modes are shown. Cell migration tracks are highlighted as computed cell centroid displacement (*yellow tracks*). Frame frequency (*i.e.*, frames per second, fps) is 32 fps, and each frame corresponds to 1 minute of real-time microscopy. The scale bar is 20 μm on the panels with denoted tracks. The scale bar on the zoom-in panels with stationary cell centroids is 50 μm.

Movie 12. Live video-sequences for MCF-7 cell migration along the soft (G′=8.6 kPa, *left*) and rigid (G′=55 kPa, *right*) collagen type-1 grids. The control conditions (+DMSO*, top*) and low actomyosin contractility (+Blebb*, bottom*) cell migration states are shown. Cell migration tracks are highlighted as computed cell centroid displacement (*yellow tracks*). See cell detachment in (+Blebb+DA on G′=55 kPa) in Movie 5. Frame frequency (*i.e.*, frames per second, fps) is 32 fps, and each frame corresponds to 1 minute of real-time microscopy. The scale bar is 20 μm on the panels with denoted tracks. The scale bar on the zoom-in panels with stationary cell centroids is 50 μm.

Movie 13. 3D microscopy reconstruction of soft (G′ ≈ 8.9 ± 0.6 kPa) GHS scaffold (interstitial space is green).

Movie 14. 3D microscopy reconstruction of stiff (G′ ≈ 52.2 ± 4.8 kPa) GHS scaffold (interstitial space is green).

SUPPORTING INFORMATION

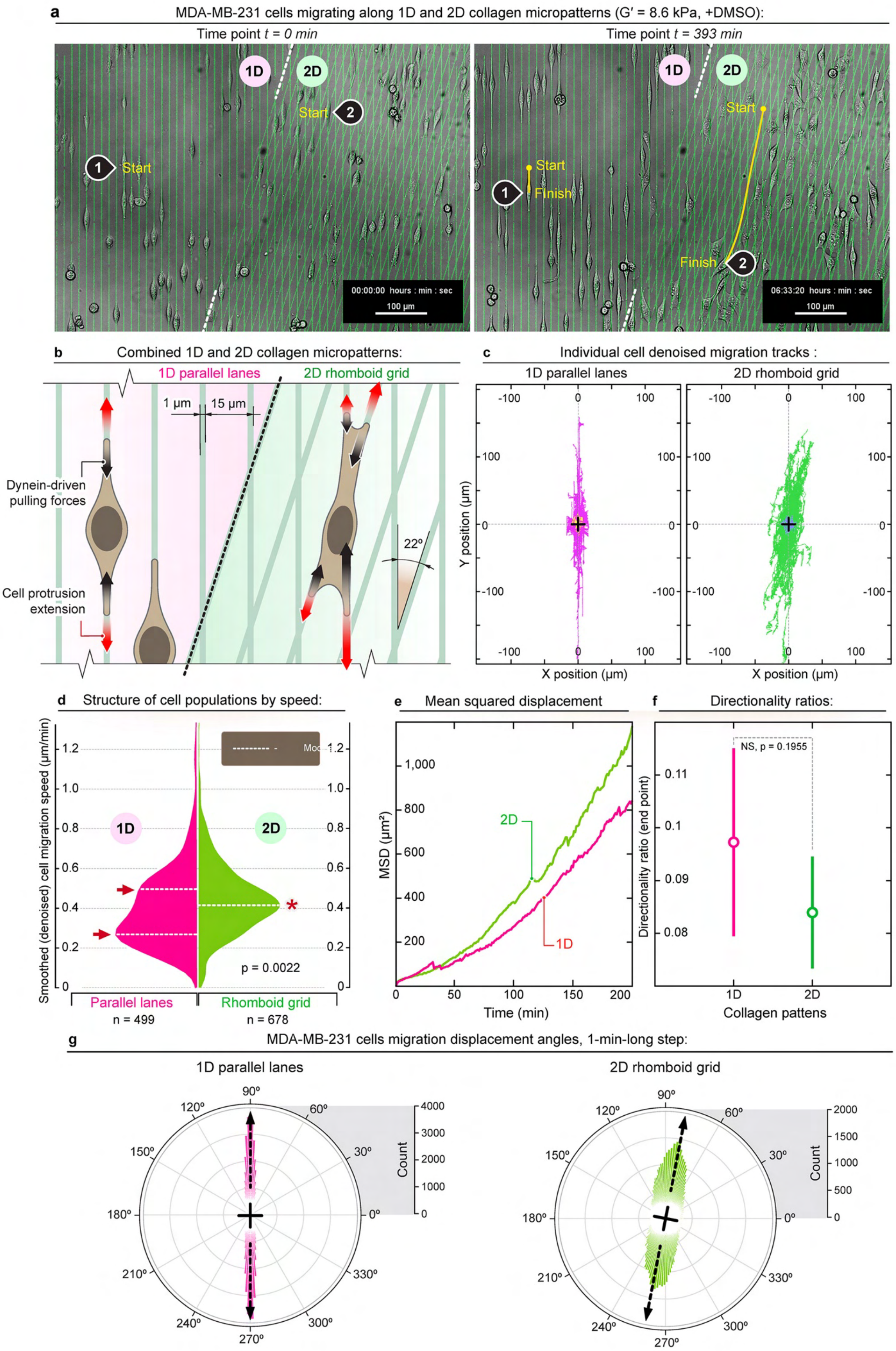
a
MDA-MB-231 cells migrating along 1D and 2D collagen micropatterns (G′ = 8.6 kPa, +DMSO):
Time point t = 0 min
Time point t = 393 min
1D
2D
Start
Finish
00:00:00 hours : min : sec
06:33:20 hours : min : sec
100 μm
b
Combined 1D and 2D collagen micropatterns:
1D parallel lanes
2D rhomboid grid
1 μm
15 μm
Dynein-driven pulling forces
Cell protrusion extension
22º
c
Individual cell denoised migration tracks :
1D parallel lanes
2D rhomboid grid
Y position (μm)
X position (μm)
d
Structure of cell populations by speed:
Smoothed (denoised) cell migration speed (μm/min)
p = 0.0022
Parallel lanes
n = 499
Rhomboid grid
n = 678
e
Mean squared displacement
MSD (μm²)
Time (min)
f
Directionality ratios:
NS, p = 0.1955
Directionality ratio (end point)
Collagen pattens
g
MDA-MB-231 cells migration displacement angles, 1-min-long step:
1D parallel lanes
2D rhomboid grid
Count

Supplementary Figure S1. Comparison of MDA-MB-231 cells' 1D migration along the parallel collagen lanes *vs*. 2D migration along the collagen rhomboid grid.

(a) Combined field of view of the brightfield and fluorescence microscopy images of the MDA-MB-231 cells migrating along the collagen lane (*left*) and grid (*right*) micropatterns (G′ =8.6 kPa) at the beginning (t=0 min) and the end of the time-lapse experiment (t=393 min). *Note the highlighted cell displacement tracks along the 1D and 2D collagen micropatterns (yellow tracks).*

(b) Schematic representation of MDA-MB-231 cell morphology and forces on micropatterns. *Note that on the collagen lanes, MDA-MB-231 cells acquire symmetric spindle-like shapes often resulting in symmetrically configured counterbalancing pulling forces and stalled migration. Other cells that acquire polarized protrusive activity can move efficiently along their 1D track until colliding with another cell on the same lane.*

(c) Individual cell 1D migration tracks on lanes (*left*) and 2D migration tracks on rhomboid grids (*right*).

(d) Population-wide distribution of migration speeds of MDA-MB-231 cells along the collagen lanes (*left*) and rhomboid grids (*right*). *Note that 1D migration speeds for cells on collagen lanes form a bi-modal distribution with modes (arrows) both below and above the single migration speed mode displayed by cells on the collagen rhomboid grid (asterisk).*

(e) Mean squared displacement indicates a higher population-wide efficiency of MDA-MB-231 cell migration on the collagen rhomboid grid compared to the 1D migration on the collagen lanes.

(f) Directionality ratio indicates a less confined (more circumnavigating) MDA-MB-231 cell migration on the collagen rhomboid grid compared to the 1D migration on the collagen lanes.

(g) Direction distributions of cell migration on the 1D parallel lanes and 2D rhomboid grid micropatterns. *Note the more frequent cell displacement events in the direction transverse to the axis of collagen patterns for the cells on 2D collagen rhomboid grids, compared to the cells on 1D collagen lanes patterns.*

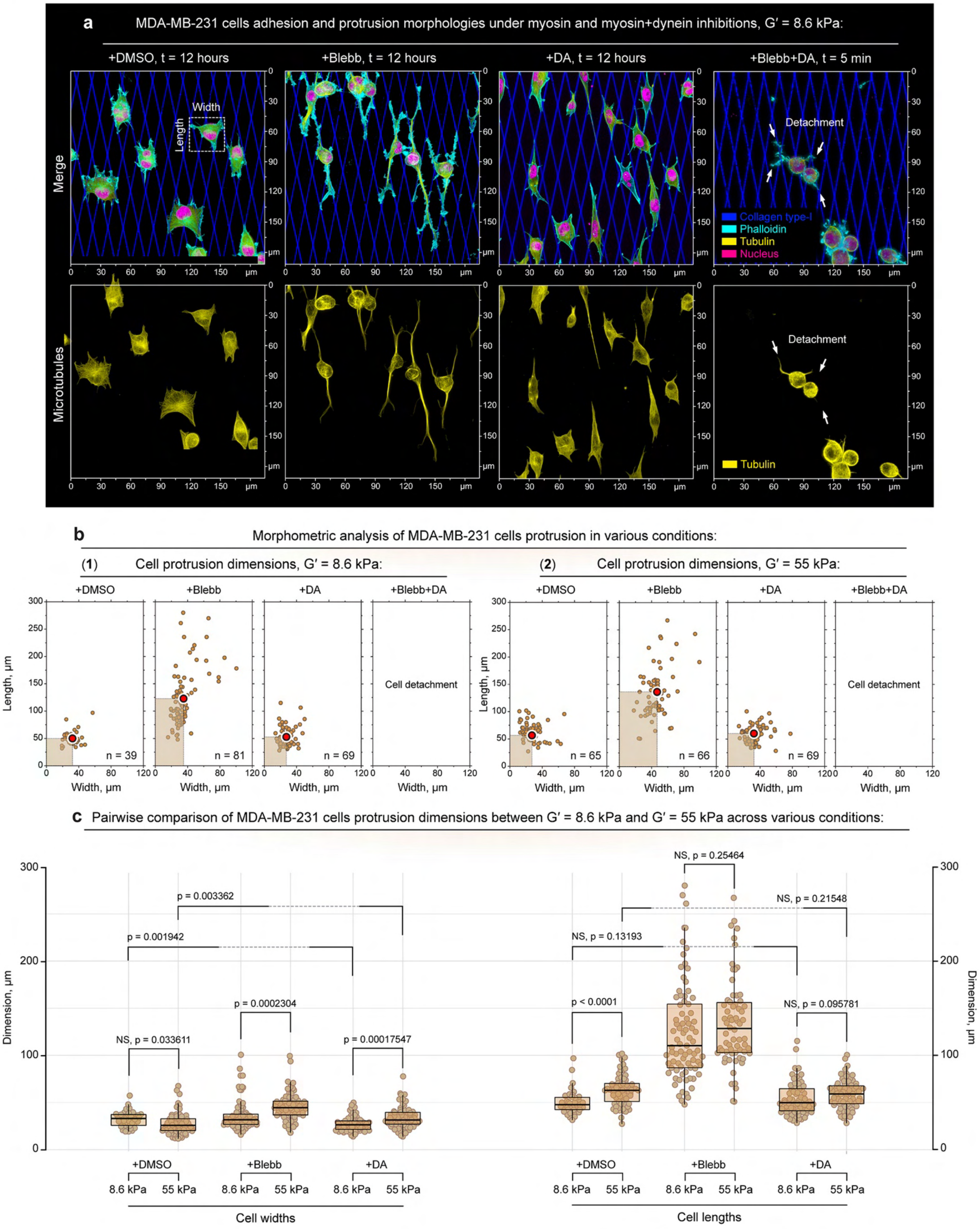
a
MDA-MB-231 cells adhesion and protrusion morphologies under myosin and myosin+dynein inhibitions, G′ = 8.6 kPa:
+DMSO, t = 12 hours
+Blebb, t = 12 hours
+DA, t = 12 hours
+Blebb+DA, t = 5 min
Merge
Microtubules
Width
Length
Detachment
Collagen type-I
Phalloidin
Tubulin
Nucleus
μm
b
Morphometric analysis of MDA-MB-231 cells protrusion in various conditions:
(1) Cell protrusion dimensions, G′ = 8.6 kPa:
(2) Cell protrusion dimensions, G′ = 55 kPa:
+DMSO
+Blebb
+DA
+Blebb+DA
Cell detachment
n = 39
n = 81
n = 69
n = 65
n = 66
n = 69
Length, μm
Width, μm
c
Pairwise comparison of MDA-MB-231 cells protrusion dimensions between G′ = 8.6 kPa and G′ = 55 kPa across various conditions:
Dimension, μm
p = 0.003362
p = 0.001942
p = 0.0002304
NS, p = 0.033611
p = 0.00017547
NS, p = 0.25464
NS, p = 0.21548
NS, p = 0.13193
p < 0.0001
NS, p = 0.095781
+DMSO
+Blebb
+DA
8.6 kPa
55 kPa
Cell widths
Cell lengths

Supplementary Figure S2. Morphometric analysis of cell protrusive spreading on the collagen-1 rhomboid grid for MDA-MB-231 cells subjected to the alternate and combined inhibition of dynein and non-muscle myosin II motors.

(a) Immunofluorescence images of MDA-MB-231 cells upon 12-hour-long attachment and protrusive spreading atop the soft (G′=8.6 kPa) collagen rhomboid grids in control conditions (+DMSO), during non-muscle myosin II suppression (+Blebb), during dynein inhibition (+DA), and during combined treatment (+Blebb+DA).

(b) Morphometric analysis of cell protrusive spreading dimensions as shown on panel **(a)**. Subpanels (**1**) and (**2**) correspond to the cells protruding on the soft (G′=8.6 kPa) and rigid (G′=55 kPa) collagen rhomboid grids, respectively. The mean values for cell dimensions are outlined with a rectangle on each corresponding plot.

(c) Pairwise comparison of cell lengths and widths between cells on soft and rigid grids.

Data are shown as box and whisker diagrams: first quartile, median, third quartile, and 95% percent confidence interval.

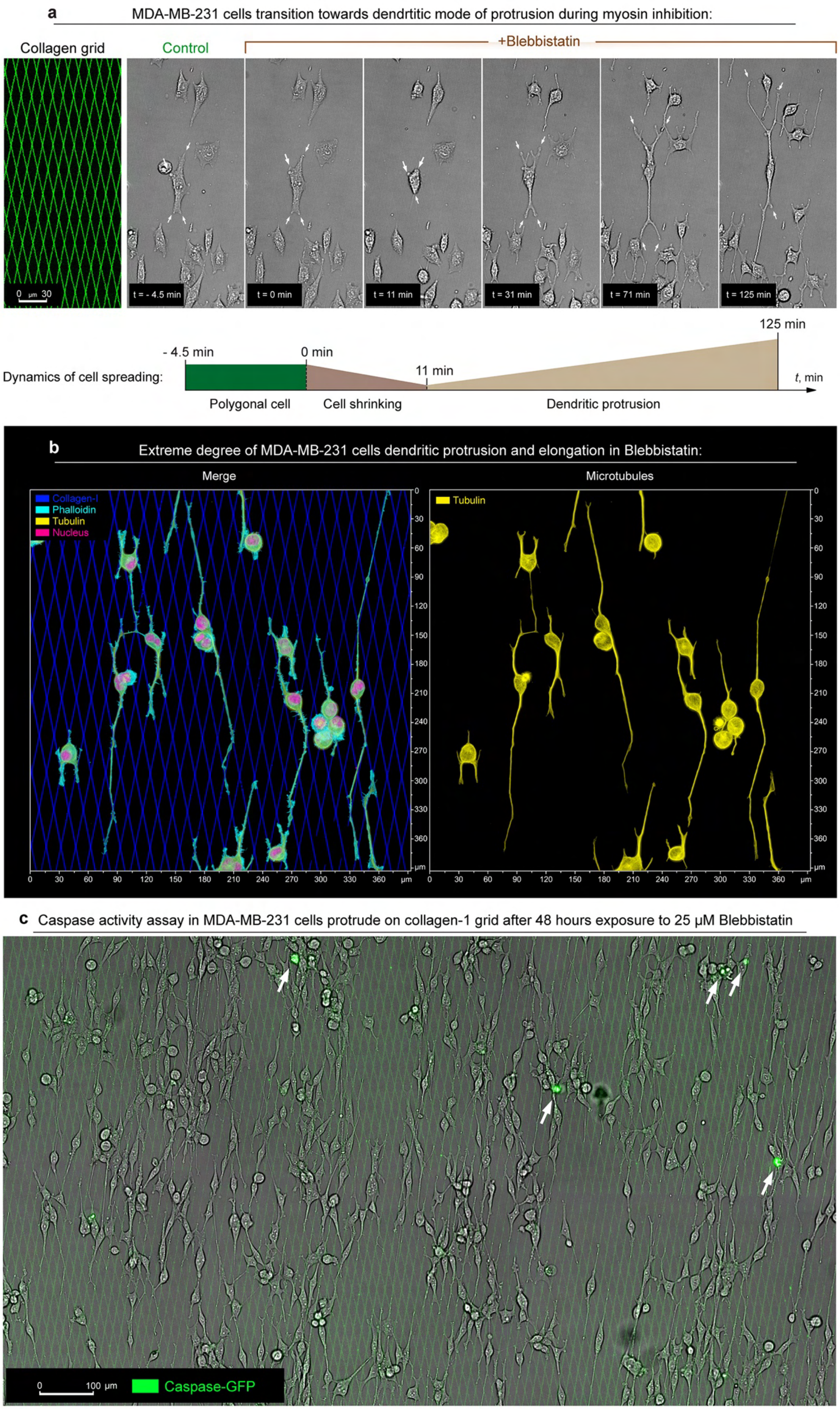
a
MDA-MB-231 cells transition towards dendrtitic mode of protrusion during myosin inhibition:
Collagen grid
Control
+Blebbistatin
0 μm 30
t = - 4.5 min
t = 0 min
t = 11 min
t = 31 min
t = 71 min
t = 125 min
Dynamics of cell spreading:
- 4.5 min
0 min
11 min
125 min
t, min
Polygonal cell
Cell shrinking
Dendritic protrusion
b
Extreme degree of MDA-MB-231 cells dendritic protrusion and elongation in Blebbistatin:
Merge
Microtubules
Collagen-I
Phalloidin
Tubulin
Nucleus
Tubulin
0 30 60 90 120 150 180 210 240 270 300 330 360 μm
c
Caspase activity assay in MDA-MB-231 cells protrude on collagen-1 grid after 48 hours exposure to 25 μM Blebbistatin
0 100 μm
Caspase-GFP

Supplementary Figure S3. Analysis of MDA-MB-231 cell viability during 'dendritic'-like protrusive spreading and migration.

(a) Control MDA-MB-231 cells grown overnight on collagen grids (G′=55 kPa) display a biphasic transition from the myosin-dependent 'polygonal' morphology towards the myosin-independent (+Blebb) 'dendritic'-like morphology. Upon addition of blebbistatin, polygonal MDA-MB-231 cells initially display a transient phase of partial detachment and protrusion span shrinkage. However, cells quickly resume protrusion (t = 11 min) in a myosin-independent 'dendritic'-like manner that results in a greater span of protrusions (*arrows*) along the collagen-1 grid.

(b) Prolonged 'dendritic'-like MDA-MB-231 cell protrusion in blebbistatin (t = 48 hours) often results in an extreme degree of cell 'dendrites' elongation.

(c) Suspended MDA-MB-231 cells were seeded on collagen grids (G′=55 kPa) in the presence of blebbistatin and CellEvent™ Caspase-3/7 detection reagent. Blebbistatin-treated suspended cells attach to the substrate and demonstrate myosin-independent 'dendritic'-like protrusion and migration. Analysis of cell viability during an extended blebbistatin treatment period (t = 48 hours) shows a negligible (≤1% of the cell population) level of apoptotic cells, as demonstrated by the Caspase-3/7 signal (arrows).

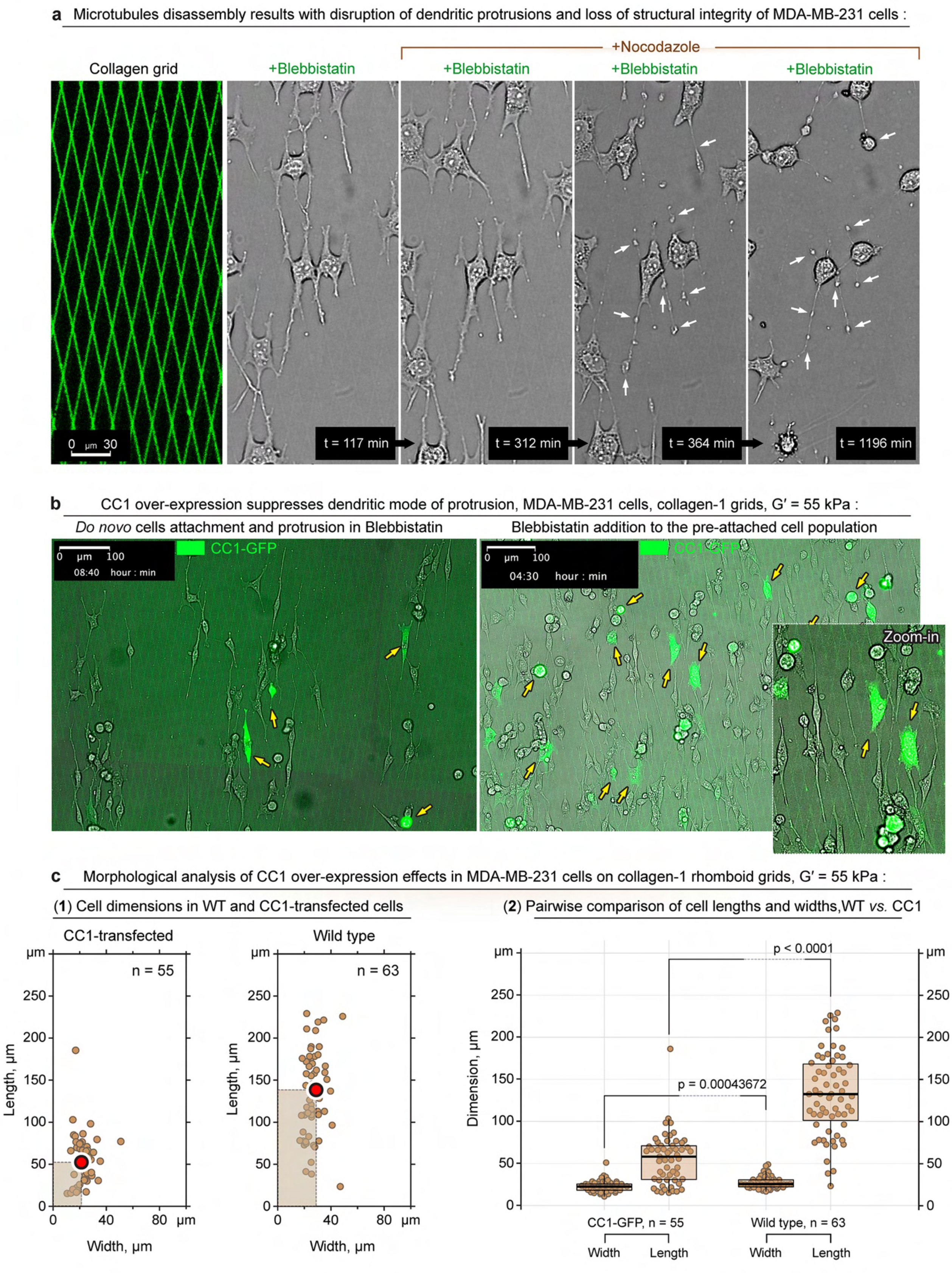

a Microtubules disassembly results with disruption of dendritic protrusions and loss of structural integrity of MDA-MB-231 cells :
+Nocodazole
Collagen grid
+Blebbistatin
+Blebbistatin
+Blebbistatin
+Blebbistatin
0 μm 30
t = 117 min
t = 312 min
t = 364 min
t = 1196 min
b CC1 over-expression suppresses dendritic mode of protrusion, MDA-MB-231 cells, collagen-1 grids, G′ = 55 kPa :
Do novo cells attachment and protrusion in Blebbistatin
Blebbistatin addition to the pre-attached cell population
0 μm 100
08:40 hour : min
CC1-GFP
0 μm 100
04:30 hour : min
Zoom-in
c Morphological analysis of CC1 over-expression effects in MDA-MB-231 cells on collagen-1 rhomboid grids, G′ = 55 kPa :
(1) Cell dimensions in WT and CC1-transfected cells
CC1-transfected
Wild type
n = 55
n = 63
Length, μm
Width, μm
(2) Pairwise comparison of cell lengths and widths,WT vs. CC1
p < 0.0001
p = 0.00043672
Dimension, μm
CC1-GFP, n = 55
Wild type, n = 63
Width
Length
Width
Length

Supplementary Figure S4. Interfering with the microtubule-dynein-dynactin system in metastatic MDA-MB-231 cells on the collagen rhomboid grid during inhibited actomyosin contractility.

(a) Microtubule disassembly with nocodazole in blebbistatin-treated MDA-MB-231 cells (nocodazole added at t = 312 min) results in disruption of dendritic protrusions and loss of structural cell integrity. Timelapse is shown in Movie 6. G′=8.6 kPa.

(b) Over-expression of the CC1 domain of p150 (Glued), which is the dynein-binding domain of the dynactin complex, suppresses ‘dendritic’-like protrusion of blebbistatin-treated cells. Note the loss of the ‘dendritic’-like mode of cell protrusion in both the *de novo* seeded MDA-MB-231 cells (*left*, Movie 7) and in the pre-attached and pre-spread MDA-MB-231 cell population (*right*, Movie 8). As a precaution, the cytoplasmic GFP marker was expressed using a separate promoter to prevent interference with the untagged CC1 domain structure, folding, or biological activity.

(c) Morphological analysis of cell dimensions for blebbistatin-treated WT and CC1-expressing MDA-MB-231 cells **(1)** and pairwise comparison of cell lengths and widths **(2)**.

Data are shown as box and whisker diagrams: first quartile, median, third quartile, and 95% percent confidence interval.

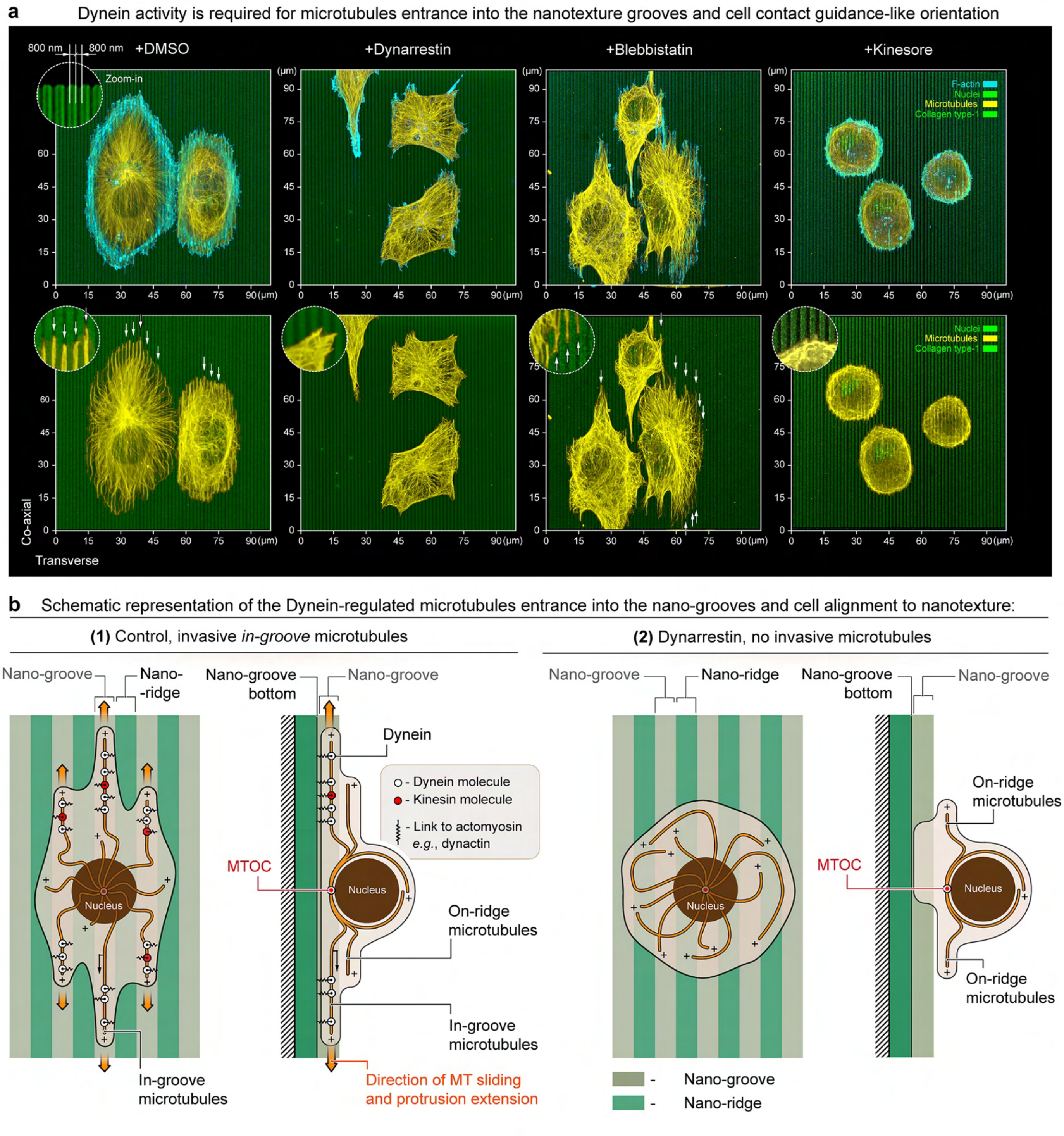

Supplementary Figure S5. Dynein activity allows microtubules entrance into the collagen type-I-functionalized nano-grooves and contact-guidance-like alignment of MDA-MB-231 cells to nanotextured surfaces.

(a) Immunofluorescence images of MDA-MB-231 cells after 12-hour-long attachment, spreading, and protrusion atop the nano-textured surface (*green*, polyurethane), functionalized with monomeric collagen-1. Cells are shown

in control conditions (+DMSO), as well as during continuous presence of dynarrestin, blebbistatin or kinesore. Nano-texture is presented by 800 nm-wide ridges, 800 nm-wide and 600 nm-deep grooves.

Top row - Visualization of F-actin (*cyan*, phalloidin) and the microtubules (*yellow*, YL/2 antibody clone).

Bottom row - Visualization of microtubule invasion into the nano-grooves in control conditions (+DMSO, *arrows*) compared to microtubules behavior during presence of blebbistatin, dynarrestin, or kinesore. Suppression of the non-muscle myosin II activity does not affect MTs' ability to enter the nano-grooves (+Blebb, *arrows*). Inhibition of the dynein (+DA) completely suppresses MTs' entrance into the nano-grooves at the cell edge, but does not affect cells' adhesion to the nano-textured collagen substrates. Overactivation of kinesin-1 with kinesore results in the MTs reorganization into the circular bands (rings), and substantial loss of the MTs in the nano-grooves.

(b) Suggested schematic representation of the dynein-regulated entrance of microtubules into the nano-grooves.

(**1**) - Dynein motors acting in the nano-grooves actively pull and align the microtubules along the adhesive cell-substrate interface, forming a network of the invasive *in-groove* microtubules that assist the structural alignment of the cell to the nanotexture direction.

(**2**) - Inhibition of the dynein motor activity with dynarrestin leads to the loss of the *in-groove* microtubules, particularly within the peripheral spreading brims of the MDA-MB-231 cells.

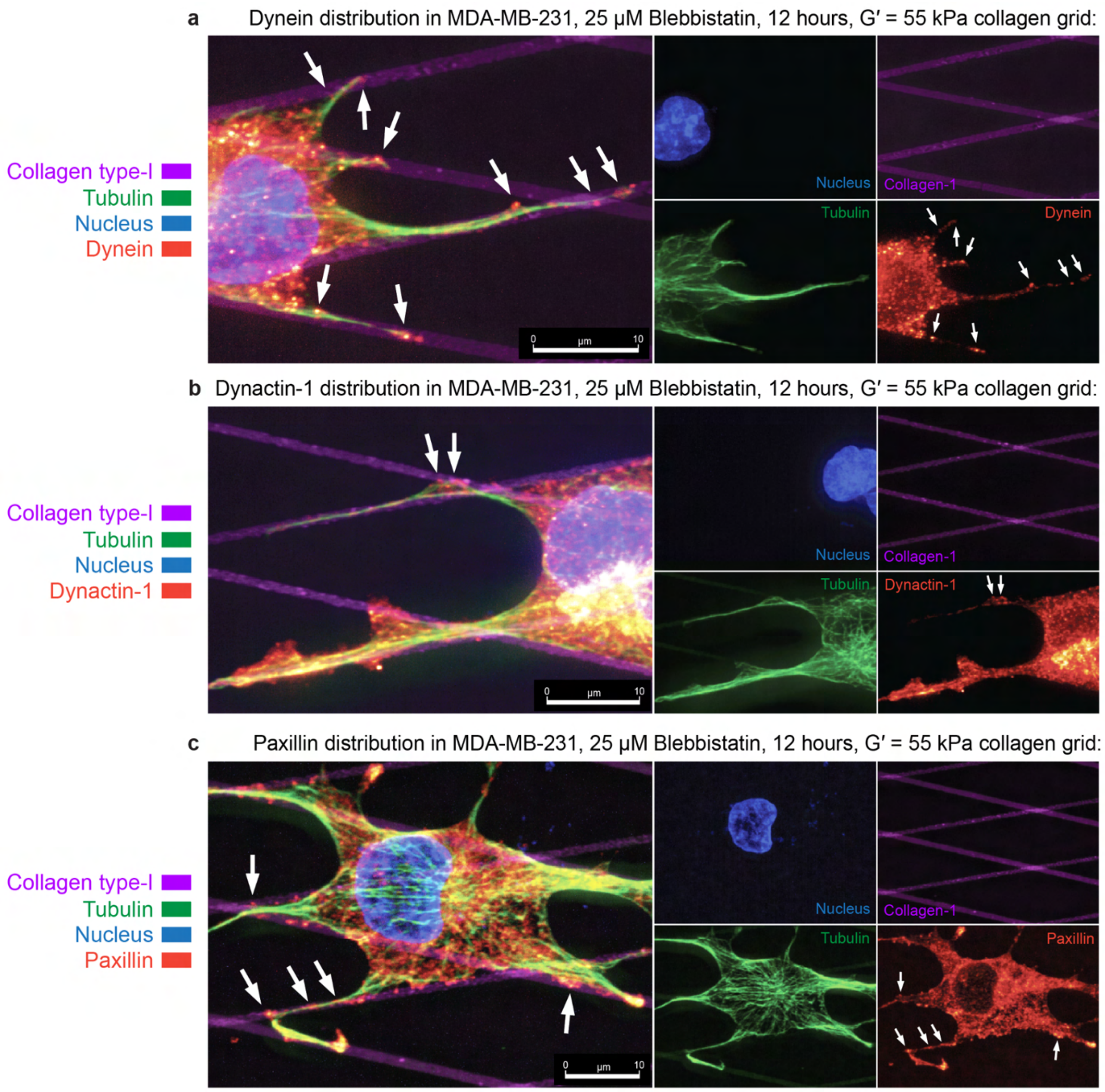

Supplementary Figure S6. Examples of subcellular distribution for Dynein, Paxillin, and Dynactin-1 during dynein-dependent migration on the collagen rhomboid grid upon inhibition of actomyosin contractility in metastatic MDA-MB-231 cells.

(a) Typical dynein distribution (red color) in MDA-MB-231 cell, upon inhibition of the actomyosin contractility (+Blebb). **(b)** Typical dynactin-1 distribution (red color) in MDA-MB-231 cell, upon inhibition of the actomyosin contractility (+Blebb). **(c)** Typical paxillin distribution (red color) in MDA-MB-231 cell, upon inhibition of the actomyosin contractility (+Blebb). *Note that all images were acquired using the instant Structured Illumination super-resolution Microscopy (iSIM) system. Cells were grown on collagen rhomboid grids (G′ = 55 kPa) for 12 hours in the presence of blebbistatin. Images represent maximum intensity projections of collected Z-stacks. Scale bars are 10 μm.*

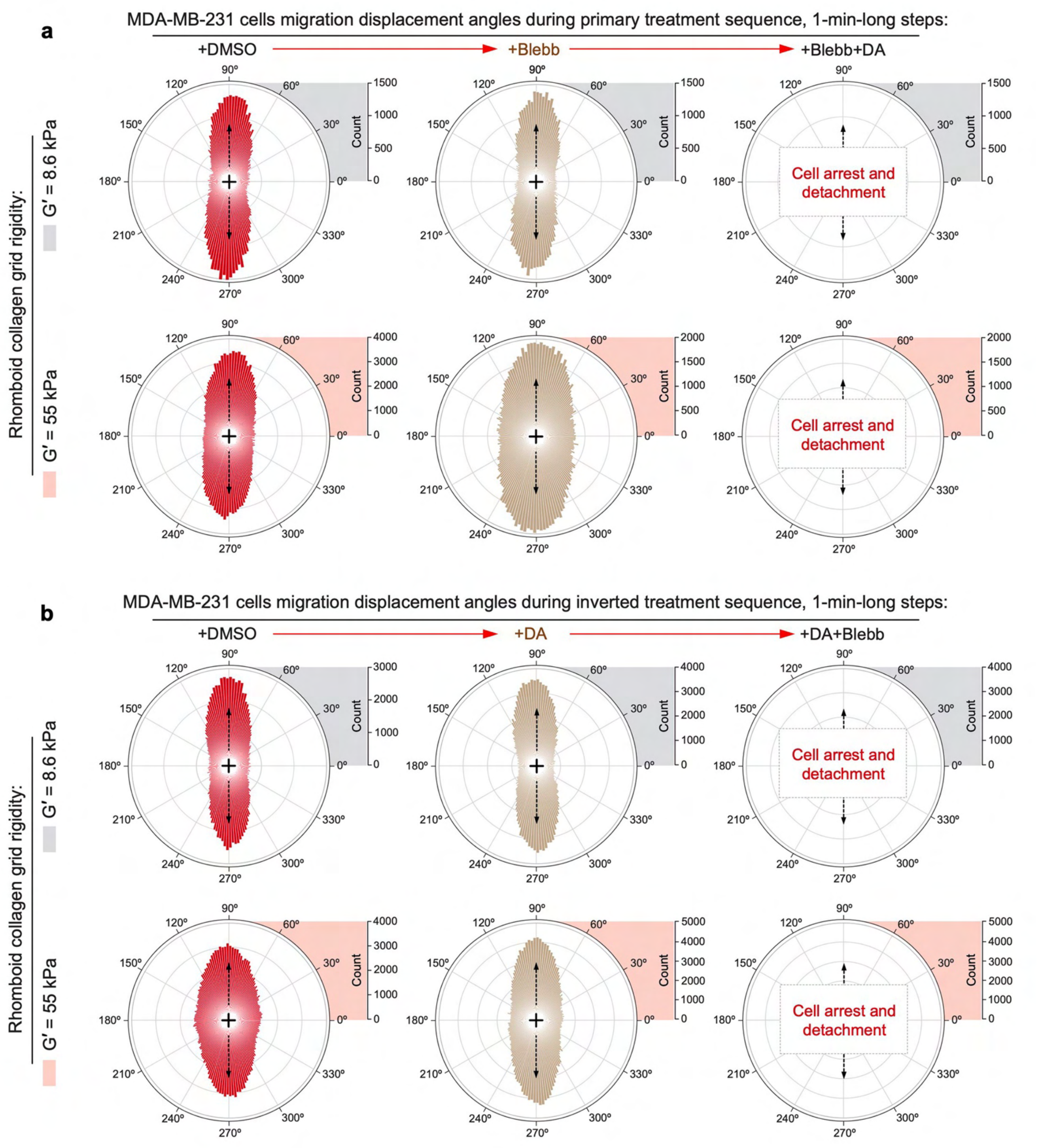

Supplementary Figure S7. Distribution of angular deflections for cell migration from the anisotropy axis of the collagen rhomboid grid for metastatic MDA-MB-231 and non-metastatic MCF-7 breast cancer cell lines under various conditions. Cumulative direction distributions of individual cell displacements are calculated for 1-minute-long timeframe steps. Displacement directions are compiled into circular diagrams with the grid anisotropy axis oriented vertically (*arrows*).

(a) Direction distributions of MDA-MB-231 cells migrating in control conditions (+DMSO, *left column*), followed by the actomyosin contractility inhibition (+Blebb, *central column*), and concluded by dynein co-suppression *via* adding dynarrestin to the blebbistatin-treated cells (+Blebb+DA, *right column*), on soft (G′ = 8.6 kPa, *top row*) and rigid (G′ = 55 kPa, *bottom row*) rhomboid collagen grids.
(b) Direction distributions of MDA-MB-231 cell displacements during inverted treatment sequence: in control conditions (+DMSO, *left*), followed by dynein activity inhibition (+DA, *center*), and with subsequent actomyosin contractility co-inhibition along with continued dynein suppression (+DA+Blebb, *right*).

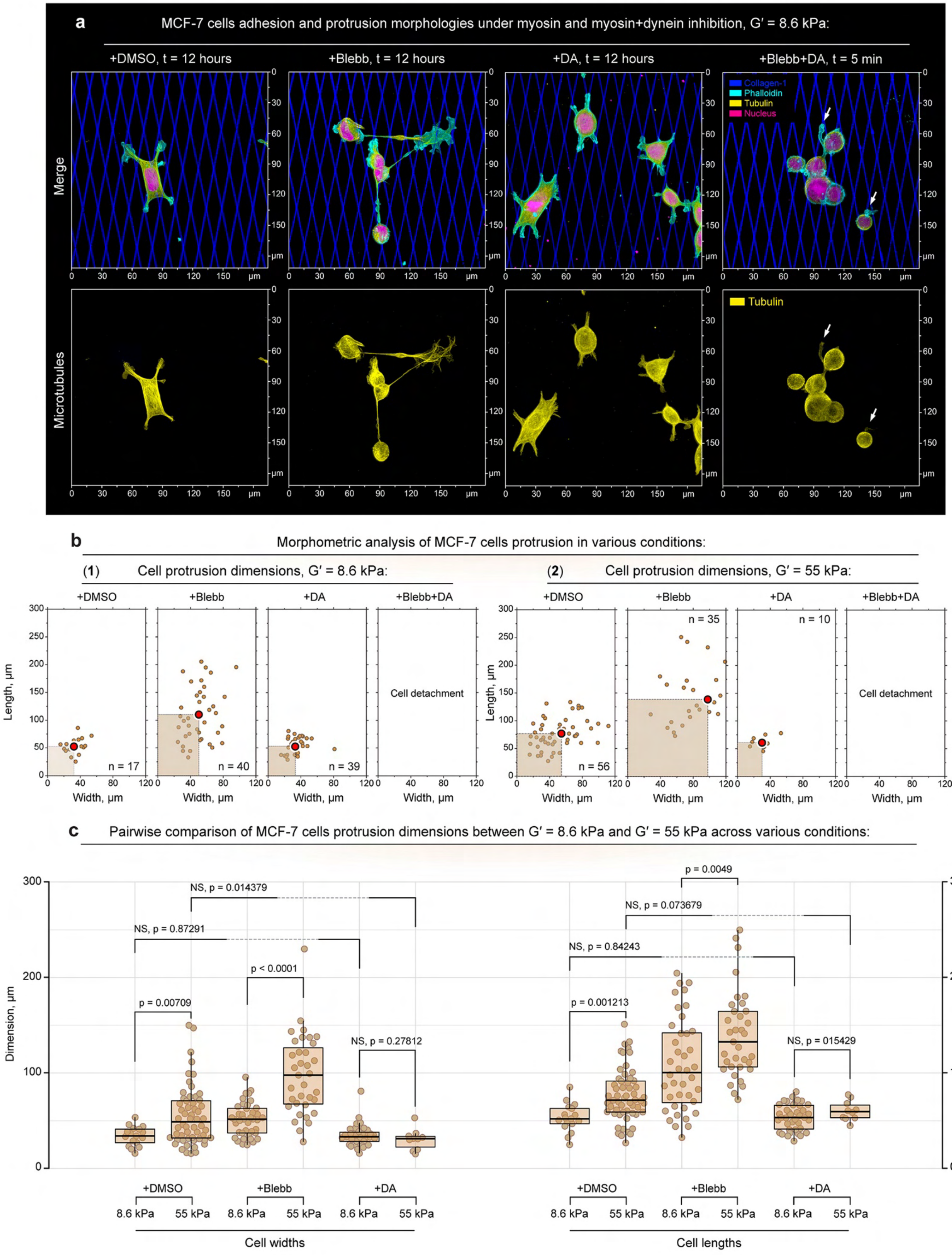

a
MCF-7 cells adhesion and protrusion morphologies under myosin and myosin+dynein inhibition, G′ = 8.6 kPa:
+DMSO, t = 12 hours
+Blebb, t = 12 hours
+DA, t = 12 hours
+Blebb+DA, t = 5 min
Collagen-1
Phalloidin
Tubulin
Nucleus
Merge
Microtubules
Tubulin
μm
b
Morphometric analysis of MCF-7 cells protrusion in various conditions:
(1) Cell protrusion dimensions, G′ = 8.6 kPa:
(2) Cell protrusion dimensions, G′ = 55 kPa:
+DMSO
+Blebb
+DA
+Blebb+DA
Length, μm
Width, μm
n = 17
n = 40
n = 39
Cell detachment
n = 56
n = 35
n = 10
Cell detachment
c
Pairwise comparison of MCF-7 cells protrusion dimensions between G′ = 8.6 kPa and G′ = 55 kPa across various conditions:
Dimension, μm
NS, p = 0.014379
NS, p = 0.87291
p < 0.0001
p = 0.00709
NS, p = 0.27812
p = 0.0049
NS, p = 0.073679
NS, p = 0.84243
p = 0.001213
NS, p = 015429
+DMSO
+Blebb
+DA
8.6 kPa
55 kPa
Cell widths
Cell lengths

Supplementary Figure S8. Morphometric analysis of MCF-7 cells' protrusive spreading on the collagen-1 rhomboid grid for cells subjected to the alternate and combined inhibition of dynein and non-muscle myosin II motors.

(a) Immunofluorescence images of MCF-7 cells upon 12-hour-long attachment and protrusion atop the soft (G′=8.6 kPa) collagen rhomboid grids in control conditions (+DMSO), during non-muscle myosin II suppression (+Blebb), during dynein inhibition (+DA), and during combined treatment (+Blebb+DA).

(b) Morphometric analysis of the dimensions of cells' protrusive spreading as shown in panel **(a)**. Subpanels (**1**) and (**2**) correspond to the cells' protrusion on the soft (G′=8.6 kPa) and rigid (G′=55 kPa) collagen rhomboid grids, respectively. The mean values of cell dimensions are outlined with a rectangle on each corresponding plot.

(c) Pairwise comparison of cell lengths and widths between cells on soft and rigid grids.

Data are shown as box and whisker diagrams: first quartile, median, third quartile, and 95% percent confidence interval.

Flow cytometry-based precision-level comparison of *per cell* Dynactin-1 expression in MCF-7 and MDA-MB-231 lines :

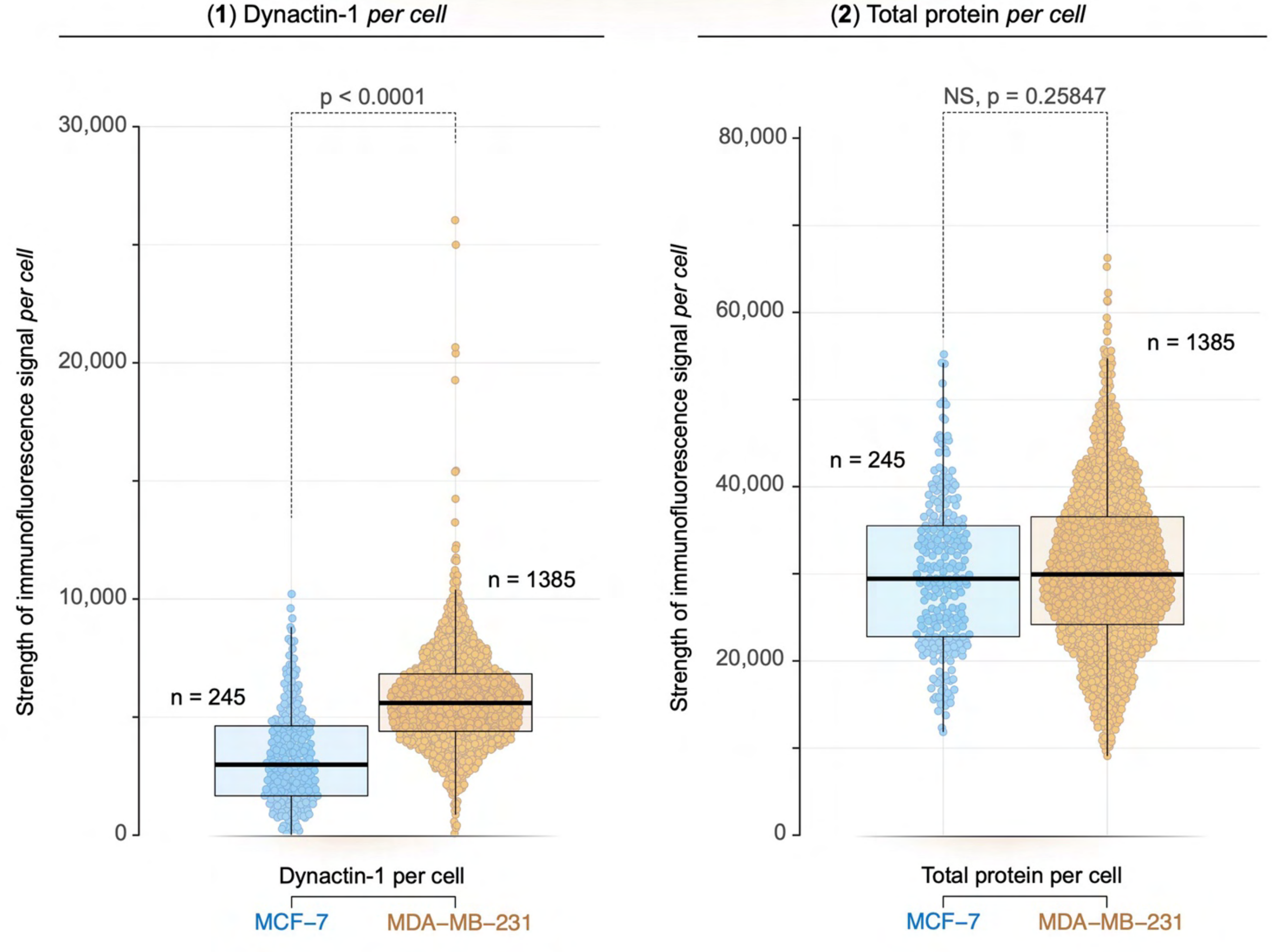

Where “n” is a confirmed single cell

Supplementary Figure S9. Comparative flow cytometry analysis of Dynactin-1 level in MDA-MB-231 and MCF-7 cell lines.

Single-cell dynactin-1 (**1**) and total protein (**2**) immunofluorescence signals were collected by flow cytometry for co-stained MDA-MB-231 (*left*, blue) and MCF-7 (*right*, brown) cells. *Note that average protein levels in individual cells are similar for both cell lines, while the average dynactin-1 level in MDA-MB-231 is approximately twice of that in MCF-7 cells.*

Data are shown as box and whisker diagrams: first quartile, median, third quartile, and 95% percent confidence interval.

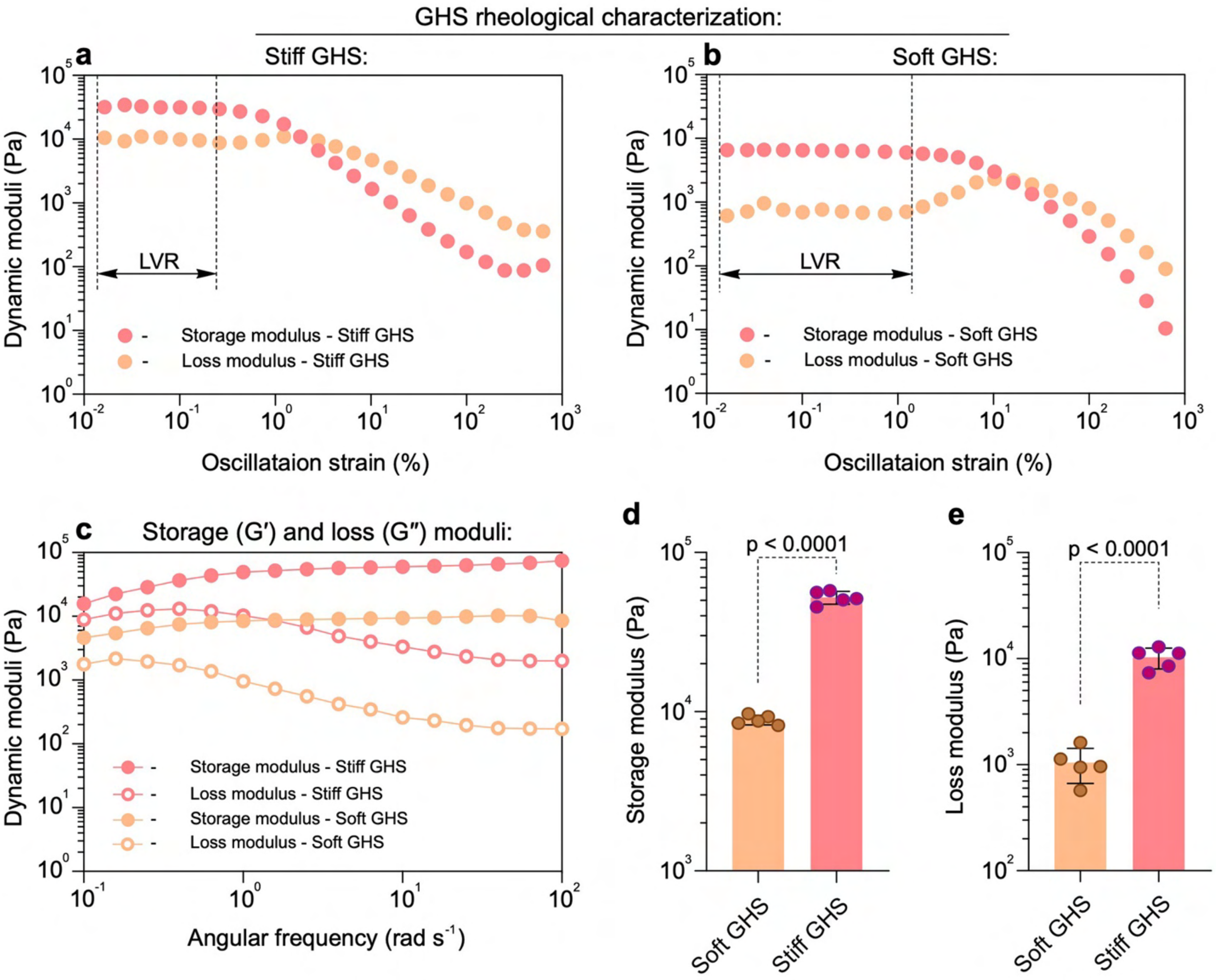

Supplementary Figure S10. Rheological characterization of GHS. Oscillation strain sweep of **(a)** stiff or **(b)** soft GHS, measured at a constant frequency of 1 rad s^{-1} to identify the linear viscoelastic region (LVR).
(c) Storage (*G′*) and loss (*G″*) moduli of soft and stiff GHS versus angular frequency, measured at an oscillatory strain of 0.1%.
(d) The average storage modulus of soft and stiff GHS, acquired at a strain of 0.1% and angular frequency of 1 rad s^{-1}.
(e) The average loss modulus of soft and stiff GHS, measured at a constant strain of 0.1% and angular frequency of 1 rad s^{-1}.

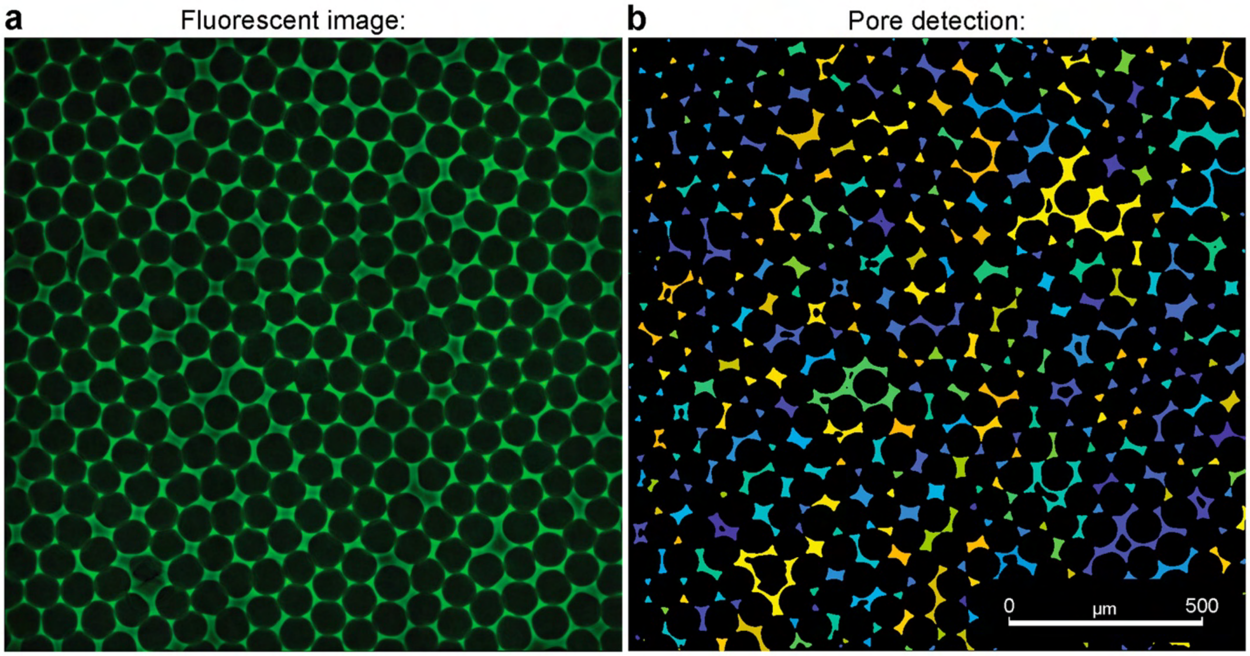

Supplementary Figure S11. **Equivalent median pore diameter analysis**. **(a)** Transversal sections of GHS based on fluorescence microscopy. **(b)** Pore detection using a user-developed MATLAB code to identify the void spaces among photochemically assembled microgels, occupied by fluorescently labeled dextran molecules (Mw = 2 MDa). To measure the equivalent pore diameter, detected pores were converted into circles with similar areas, and the median of the resulting diameter distribution was calculated.
